# A marine record of Patagonian ice sheet changes over the past 140,000 years

**DOI:** 10.1073/pnas.2302983121

**Published:** 2024-03-04

**Authors:** Julia R. Hagemann, Frank Lamy, Helge W. Arz, Lester Lembke-Jene, Alexandra Auderset, Naomi Harada, Sze Ling Ho, Shinya Iwasaki, Jérôme Kaiser, Carina B. Lange, Masafumi Murayama, Kana Nagashima, Norbert Nowaczyk, Alfredo Martínez-García, Ralf Tiedemann

**Affiliations:** ^a^Division of Geoscience, Marine Geology Section, Alfred Wegener Institute Helmholtz Centre for Polar and Marine Research, Bremerhaven 27570, Germany; ^b^Department of Climate Geochemistry, Organic Isotope Geochemistry Group, Max Planck Institute for Chemistry, Mainz 55128, Germany; ^c^Center for Marine Environmental Sciences, University of Bremen, Bremen 28359, Germany; ^d^Department of Marine Geology, Paleoceanography and Sedimentology Group, Leibniz Institute for Baltic Sea Research Warnemünde, Rostock 18119, Germany; ^e^School of Ocean and Earth Science, University of Southampton, Southampton SO17 1BJ, United Kingdom; ^f^Atmosphere and Ocean Research Institute, Center for International and Local Research Cooperation, The University of Tokyo, Kashiwa 277-8564, Japan; ^g^Research Institute for Global Change, Earth Surface System Research Center, Japan Agency for Marine-Earth Science and Technology, Yokosuka 237-0061, Japan; ^h^Institute of Oceanography, National Taiwan University, Taipei 10617, Taiwan; ^i^Graduate School of Environmental Science, Hokkaido University, Sapporo 060-0810, Japan; ^j^Departamento de Oceanografía & Centro de Investigación Oceanográfica en el Pacífico Suroriental (Coastal), Universidad de Concepción, Concepción 4030000, Chile; ^k^Centro de Investigación Dinámica de Ecosistemas Marinos de Altas Latitudes, Universidad Austral de Chile, Valdivia 5110566, Chile; ^l^Scripps Institution of Oceanography, University of California San Diego, La Jolla, CA 92037, United States; ^m^Faculty of Agriculture and Marine Science, Kochi University, Nankoku, Kochi 783-8502, Japan; ^n^Center for Advanced Marine Core Research, Kochi University, Nankoku, Kochi 783-8502, Japan; ^o^Department of Geosystems, Section of Climate Dynamics and Landscape Evolution, Helmholtz Centre Potsdam German Research Centre for Geosciences, Potsdam 14473, Germany

**Keywords:** Patagonian ice sheet, Chile, paleoceanography, continent–ocean interaction, organic biomarkers

## Abstract

Terrestrial glacial records from the Patagonian Andes and New Zealand Alps document quasi-synchronous Southern Hemisphere–wide glacier advances during the late Quaternary. However, these records are inherently incomplete. Here, we provide a continuous marine record of western–central Patagonian ice sheet (PIS) extent over a complete glacial–interglacial cycle back into the penultimate glacial (~140 ka). Sediment core MR16-09 PC03, located at 46°S and ~150 km offshore Chile, received high terrestrial sediment and meltwater input when the central PIS extended westward. We use biomarkers, foraminiferal oxygen isotopes, and major elemental data to reconstruct terrestrial sediment and freshwater input related to PIS variations. Our sediment record documents three intervals of general PIS marginal fluctuations, during Marine Isotope Stage (MIS) 6 (140 to 135 ka), MIS 4 (~70 to 60 ka), and late MIS 3 to MIS 2 (~40 to 18 ka). These higher terrigenous input intervals occurred during sea-level low stands, when the western PIS covered most of the Chilean fjords, which today retain glaciofluvial sediments. During these intervals, high-amplitude phases of enhanced sediment supply occur at millennial timescales, reflecting increased ice discharge most likely due to a growing PIS. We assign the late MIS 3 to MIS 2 phases and, by inference, older advances to Antarctic cold stages. We conclude that the increased sediment/meltwater release during Southern Hemisphere millennial-scale cold phases was likely related to higher precipitation caused by enhanced westerly winds at the northwestern margin of the PIS. Our records complement terrestrial archives and provide evidence for PIS climate sensitivity.

## Significance

Continental glaciers and ice sheets are excellent indicators of ongoing and past climate changes. The Patagonian ice sheet (PIS) was the largest extrapolar ice sheet in the Southern Hemisphere. Many studies have investigated the advances of the PIS on its eastern side, but there are only a few PIS records on the Pacific side. We show that three active intervals occurred during the last ~140 ka, with an extended PIS that contributed to the release of large amounts of freshwater and sediment into the Pacific. Active intervals during the last glacial period occurred from ~70 to 60 ka and from ~40 to 18 ka, with four and five phases of increased ice discharge, respectively, most likely driven by precipitation changes.

Investigating past ocean–atmosphere–ice interactions across an entire glacial cycle is important for assessing recent climate change through a long-term perspective and successfully predicting future climate and associated glacier changes. However, terrestrial archives are often temporally discontinuous and spatially disconnected, while marine archives suitable to study continent–ocean linkages in the Southern Hemisphere are rare (1). Patagonia describes the landscape in southern South America and hosts the northern and southern Patagonian icefields (NPI and SPI), which represent the largest continental ice masses in the midlatitudes (2). During the last glacial period, the much larger PIS extended from ~38 to 56°S (3, 4) and stored a global sea-level equivalent of up to ~1.5 m when it reached its maximum extent at ~35 ka (4, 5). The maritime location of the ice sheet along the southern Andes favored a close linkage to atmosphere–ocean changes in the southeast Pacific and the northernmost reaches of the Antarctic Circumpolar Current [ACC; (6, 7)]. However, it is still not well documented how sensitively the PIS reacted to orbital and millennial-scale changes in climate, ocean circulation, and the northward extension of the ACC.

Atmosphere–ocean–cryosphere interactions are complex along the southern Chilean continental margin (e.g., ref. 7). Atmospheric and oceanic circulation patterns off southern Chile strongly impact the supply of moisture to the Andes south of ~30°S, controlling precipitation and erosion, and consequently fluvial sediment input, vegetation, and the extent of glaciation, including the size of the PIS during glacial phases (e.g., refs. 4 and 8). Previous studies focusing on Patagonia showed that both the location and intensity of the southern westerly wind (SWW) belt played a crucial role in the formation of glaciers, and also in the supply of freshwater and sediment to the ocean (1, 4, 9).

So far, glaciological ice sheet reconstructions are available mostly from the eastern margin of the PIS (e.g., refs. 6, 7, 10, and 11–13). The pan-ice sheet empirical reconstruction [PATICE; (4)] covers the period 35 ka to present and is based on a compilation of glacial geomorphology and recalibrated chronological data across the entire ice sheet region. Maximum ice extent in the northern section (38°S to 48°S) in this reconstruction ranges from 33 ka to 28 ka, while from ~48°S southward other studies have indicated an earlier maximum extent, at 47 ka during Marine Isotope Stage (MIS) 3. The net retreat began as early as 25 ka, with ice-marginal stabilization around 21 to 18 ka, followed by rapid, irreversible deglaciation. Local PIS advances occurred earlier in the Magellan lobe (53°S), where glaciers reached full glacial extent during MIS 4 (7, 14), and further north toward the Pacific, on Chiloé Island (15). Regionally, advances at ~48°S might have already started during late MIS 5 (13). Evidence for advances in Patagonia prior to the last glacial is rare. Earlier glacier advances are only documented in northern Patagonia during MIS 6 (16–18) and central Patagonia during MIS 8 (16, 17, 19, 20), where PIS maxima extents are recorded as having been similar to those around the Last Glacial Maximum (LGM).

**Fig. 1.**
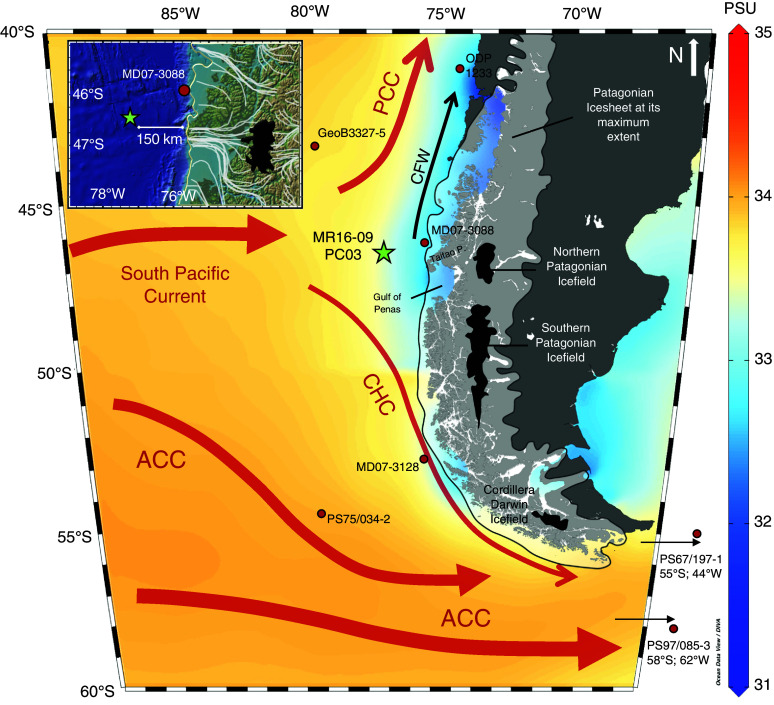
Map of the southeast Pacific and southern South America with major ocean currents and superimposed sea surface salinity [WOA13; (37)]. The maximum extent of the Patagonian ice sheet is shown in semitransparent pale shading. Black polygons mark present day Patagonian ice fields (4). Green star: site MR16-09 PC03 of this study. Red dots: marine sites discussed in the text. ACC = Antarctic Circumpolar Current; CHC = Cape Horn Current; PCC = Peru–Chile Current; CFW = Chilean Fjord Current. Insert map: Projected Ice-flow lines of PIS at 35 ka (4).

In contrast to the eastern margin of the PIS, the extent along the western side of the ice sheet toward the Pacific Ocean during the last glacial period is not well constrained (4, 5). Some ice sheet reconstructions have been carried out for the northernmost part of the Chilean lake district and on Chiloé Island [38 to 46°S; (15, 21, 22)], as well as in the southernmost part, at the Cordillera Darwin glaciers during Heinrich Stadial 1 (23). Ice sheet models (5) and seismic data (24) indicate that the PIS advanced to the marine shelf edge south of ~44°S, at least during the LGM. However, well-dated paleoenvironmental records documenting the changes in the western extent of the PIS during the last glacial are restricted to a few marine sediment records along the northern PIS margin (~38 to 46°S), and southernmost Patagonia, in the vicinity of the Pacific entrance to the Magellan Strait (~53°S). These records primarily include ODP Site 1233 (~41°S) reaching back to ~70 ka (25–27), MD07-3088 (~45°S) reaching back to ~23 ka (28–30), and MD07-3128 (~53°S) reaching back to ~60 ka (31, 32). Longer sediment records from the Chilean continental margin (Fig. 1), which cover a complete glacial–interglacial cycle, are challenging to obtain due to high terrigenous sediment input from the Andean hinterland. Thus, they are restricted to the continental margin north of the PIS off central Chile (ODP Site 1234; e.g., refs. 33 and 34) and further offshore in the southern section of the southeast Pacific [GeoB3327-5 and PS75/034-2; (35, 36)], locations which are only partially within the range of terrigenous sediment input from South America (Fig. 1).

Here, we provide results from a well-dated marine record that we link to PIS marginal fluctuations and their interaction with palaeoceanographic variations in the adjacent Southeast Pacific covering the past ~140 ka. Marine sediment core MR16-09 PC03 was retrieved at 46° 24.32′S, 77° 19.45′ W from a water depth of 3,082 m (Fig. 1). The site is located ~150 km offshore the Taitao Peninsula in southern Chile (Fig. 1) on the western flank of the Chile Rise, which is being subducted in this region. The core location is positioned above regional depressions and, therefore, mostly sheltered from turbidity currents (*SI Appendix*, Fig. S1). During interglacials, the area received little terrigenous input and sediments are predominantly biogenic, consisting mainly of nannofossil and foraminifera oozes (38). This predominantly biogenic content is in strong contrast to primarily terrigenous sedimentation during glacial intervals related to a reconstructed major outflow of the PIS [Fig. 1; PATICE reconstruction, (4)], dominated by silty clay with minor contents of diatoms and nannofossils (**SI Appendix*, Methods*).

**Fig. 2.**
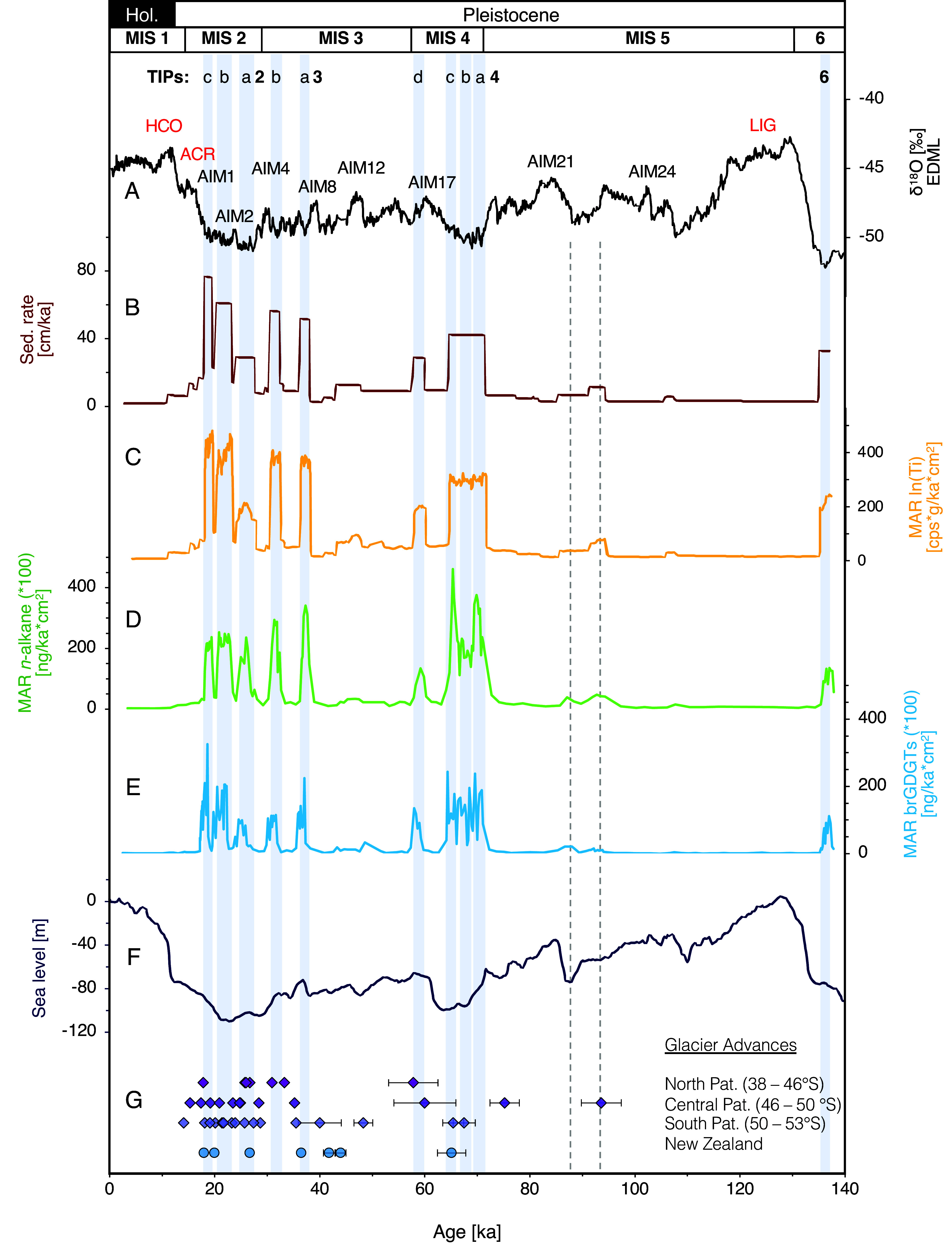
Terrigenous input proxy records from core MR16-09 PC03 over the past 140 ka. Gray stripes and numbers at the top mark Terrigenous Input Phases (TIPs). (*A*) δ^18^O record from the EDML ice core (40). HCO = Holocene Climate Optimum, ACR = Antarctic Cold Reversal, LIG = Last Interglacial, AIM = Antarctic Isotope Maxima. (*B*) Bulk sedimentation rates. (*C*) Titanium content (11-point moving average). (*D*) The mass accumulation rate of *n*-alkanes. (*E*) The mass accumulation rate of branched GDGTs. (*F*) Eustatic sea level reconstruction (41). (*G*) Individual glacier advances of Patagonia (Pat.) and New Zealand. Advances from North Patagonia were taken from refs. 15 and 22. Advances from Central Patagonia were taken from refs. 11–13. Advances from South Patagonia were taken from refs. 6, 7, 10, and 42. Advances from New Zealand were taken from refs. 43 and 44. Literature ages are found in *SI Appendix*, Table S4.

**Fig. 3.**
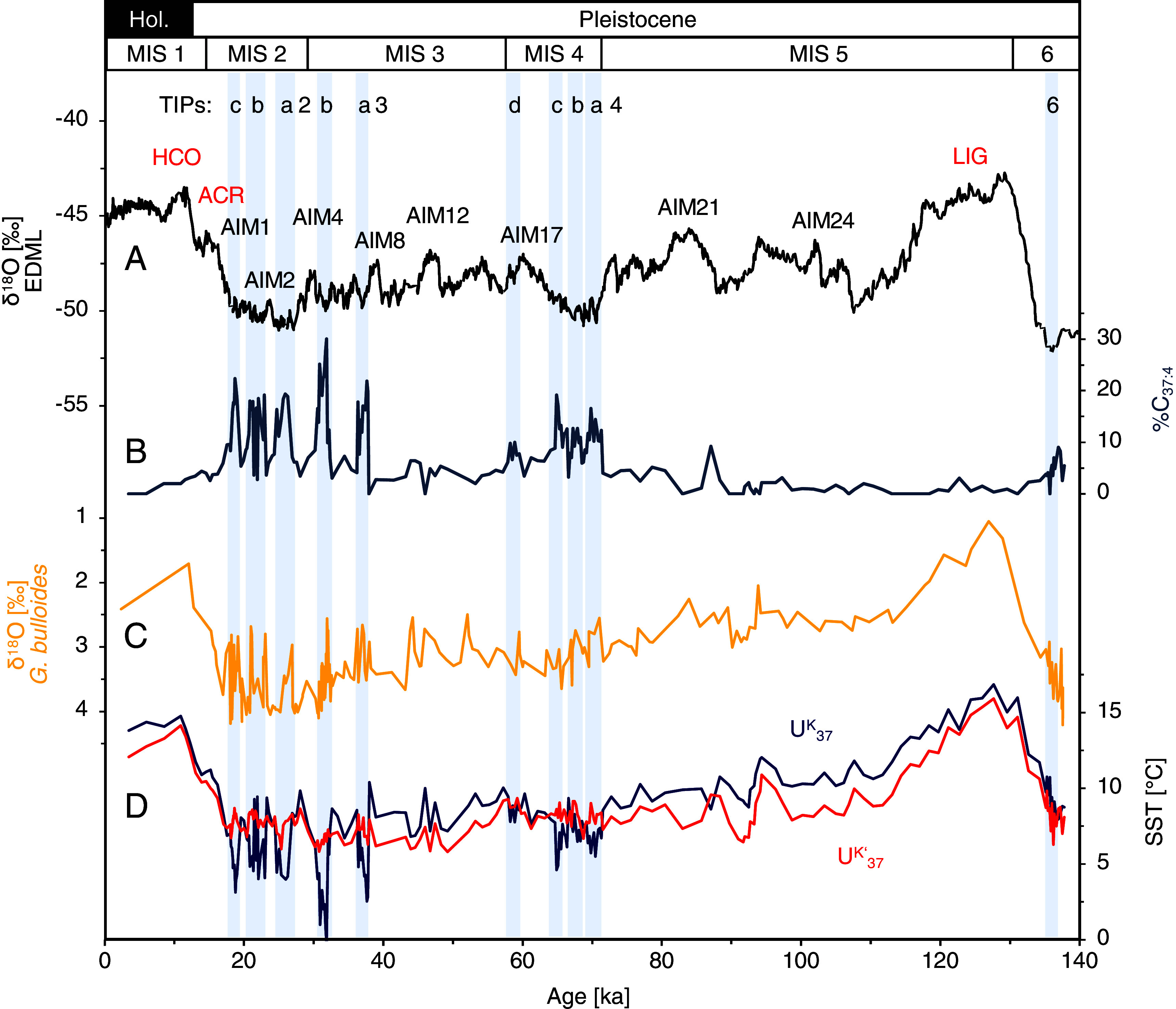
Freshwater input records from core MR16-09 PC03 over the past 140 ka. Gray stripes and numbers at the top mark Terrigenous Input Phases (TIP). (*A*) δ^18^O record from the EDML ice-core (40). HCO = Holocene Climate Optimum, ACR = Antarctic Cold Reversal, LIG = Last Interglacial, AIM = Antarctic Isotope Maxima. (*B*) Percentage of alkenone C_37:4_ as a proxy for freshwater input with increasing values indicating lower salinity. (*C*) δ^18^O of the surface-dwelling foraminifera *G. bulloides*. (*D*) Alkenone-derived SSTs, based on U^K^_37_ (blue) and U^K’^_37_ (red).

**Fig. 4.**
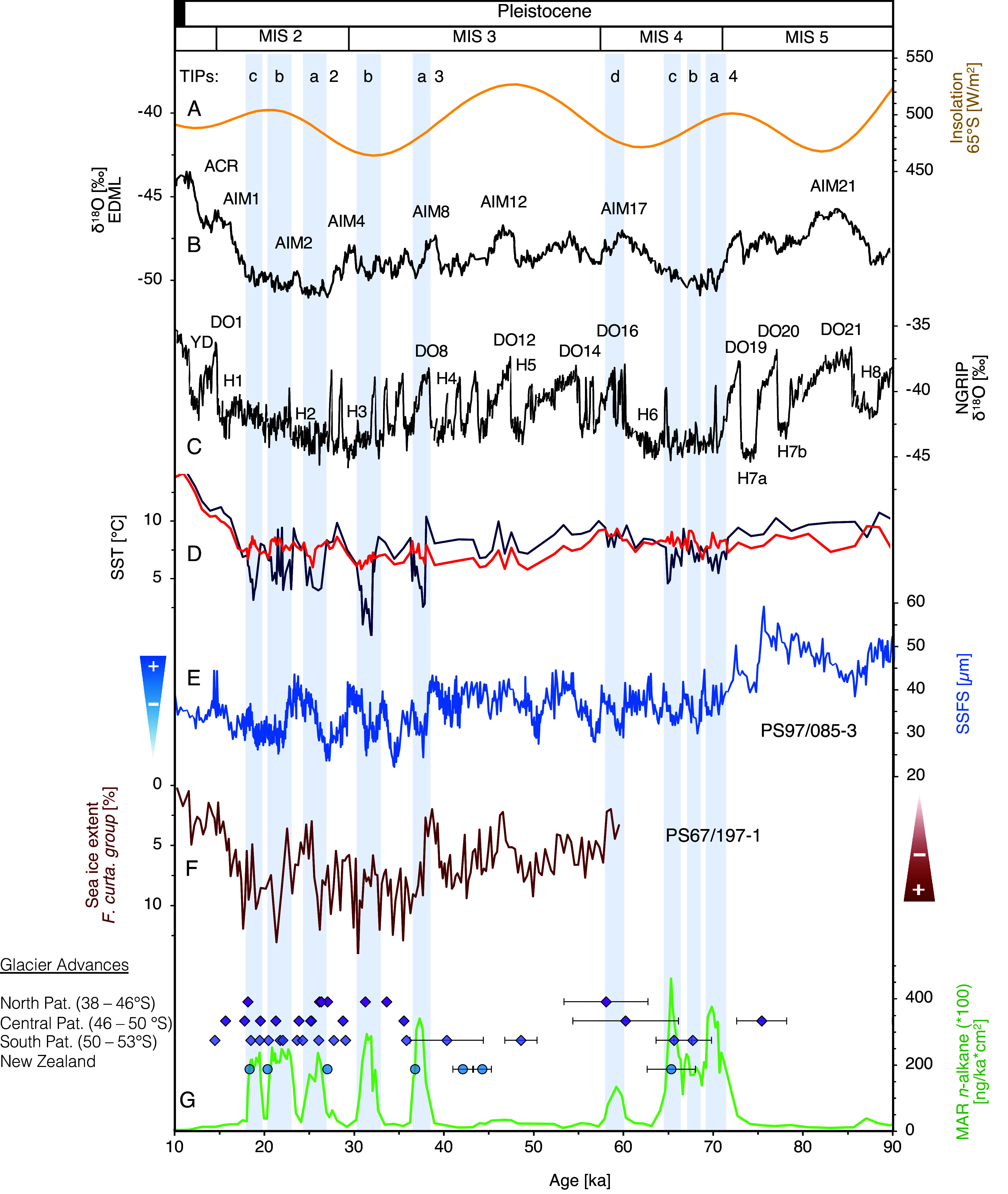
Detailed view of glacial advances between 90 and 10 ka. Gray stripes and numbers at the top mark terrigenous input phases (TIP). (*A*) SH summer insolation at 65°S in W/m^2^ (72). (*B*) δ^18^O record from the EDML ice-core (40). ACR = Antarctic Cold Reversal, LIG = Last Interglacial, AIM = Antarctic Isotope Maxima. (*C*) δ^18^O ice core NGRIP with Dansgaard–Oeschger (DO), Heinrich Events (H) and Younger Dryas [YD; (73)]. (*D*) Alkenone-derived SSTs, based on U^K^_37_–index (blue) and U^K’^_37_–index (red). (*E*) ACC strength reconstruction from the Drake Passage is based on the sortable silt/fine sand (SSFS) of core PS97/085-3 (71). (*F*) Winter sea ice extent in the Scotia Sea (PS67/197-1) based on the diatom group *Fragilariopsis curta* (71). (*G*) The mass accumulation rate of *n*-alkanes with individual glacier advances of Patagonia (Pat.) and New Zealand. Advances from North Patagonia were taken from refs. 15 and 22. Advances from Central Patagonia were taken from refs. 11–13. Advances from South Patagonia were taken from refs. 6, 7, 10, and 42. Advances from New Zealand were taken from refs. 43 and 44. Literature ages are found in *SI Appendix*, Table S4.

The initial age model of core MR16-09 PC03 is based on an assignment of Marine Isotope Stages using oxygen isotope ( δ^18^O) records from deep-dwelling foraminifera and comparison against standard δ^18^O stacks for the South Pacific (39). In particular, we used the δ^18^O record of the deep-dwelling planktic species *Globorotalia truncatulinoides,* which allows us to recognize millennial-scale structures during MIS 3. Further age-control points are based on radiocarbon dating for the past 40 ka, and the onset and termination of the Laschamps geomagnetic excursion at ~42.5 ka and 40.9 ka (*SI Appendix*, *Methods* and Figs. S2 and S3 and Tables S1 and S2). In a final step, we compared our proxies with the δ^18^O values of the EPICA Dronning Maud Land (EDML) ice core (Fig. 2*A*) of Antarctica to better understand the relationships between millennial-scale Antarctic Isotope Maxima (AIM) and re-occurring high terrigenous input phases (TIP) derived from the PIS (40). We define each TIP (gray stripes in Figs. 2–4) as intervals with a biomarker content (*n*-alkanes, brGDGTs: branched Glycerol Dialkyl Glycerol Tetraether) that is 20-fold higher than the average Holocene (*SI Appendix*, Fig. S4*A*). These maxima are accompanied by sudden sedimentation rate and titanium increases that are higher than the averaged Holocene background sedimentation by a minimum factor of 8 (*SI Appendix*, Fig. S4*B*). Stratigraphically, core MR16-09 PC03 reaches back to the terminal phase of MIS 6 and thus covers the complete last glacial–interglacial cycle into the Holocene, with an average sedimentation rate of 12 cm/ka (Fig. 2*B*). Lower rates occur during interglacials, particularly during MIS 5.5 and the Holocene. Intermediate rates are characteristic for most of MIS 5, MIS 4, and early MIS 3. Sedimentation rates are substantially higher during peak glacial intervals, i.e., late MIS 3 and MIS 2, reaching several tens of cm/ka (Fig. 2*B*).

## Terrestrial Input and a Western Extended Patagonian Ice Sheet

We reconstructed glacial changes in the western extent of the PIS by analyzing multiple terrigenous sedimentary proxies, including major element composition [i.e., titanium (Ti), which reflects siliciclastic input from Andean rocks; Fig. 2*C*], and terrestrial biomarkers (long-chain *n*-alkanes, branched GDGTs; Fig. 2 *D* and *E*). Glacial erosion, particularly in high mountain settings, is generally assumed to strongly enhance the overall glaciofluvial sediment flux (45). Reconstructed last glacial PIS flow pathways in central Patagonia at 35 ka indicate major ice discharge toward the Pacific from the area of the modern NPI and the northern tip of the SPI, along the southern coast of the Taitao Peninsula and the northern Gulf of Penas [Fig. 1; (4)]. This implies the probability of an increased sediment supply to our core site located ~150 km offshore at times when the central PIS stretched over the continental margin and is consistent with the substantially increased bulk sedimentation rates at our site during glacial periods. Hence, we assume an extended marine-based ice sheet at its western margin during glacials, with increased ice discharge during TIPs, and a decreased ice discharge between the TIPs, although it remains unclear whether this was accompanied by a retreat of the PIS. We presume that a retreat of only a few kilometers would be sufficient for a drastic decrease in offshore sediment supply, i.e., at times when most of the terrigenous input would remain within the vast Chilean fjord system (46). However, due to a limited amount of submarine geomorphological data, we cannot ascertain when the margin of the ice sheet really became marine-based and extended to the shelf edge. Therefore, we use the term “extended ice cover” to describe an extent of the PIS that is able to contribute to substantial amounts of sediment to our coring site. In combination with our site’s location directly at the modern bifurcation of the South Pacific Current, we further examined the potential sensitivity of the central PIS to changes in the ocean–atmosphere system via expansion and contraction of the SWW belt, affecting precipitation and associated changes in ocean temperatures (Fig. 1).

During the past ~140 ka, our sediment record documents three major intervals of overall extended western–central PIS on orbital timescales, with the main reconstructed outflow region at the western tip of the Taito peninsula (4). The first interval is during MIS 6 (~140 to 135 ka), followed by a second interval at MIS 4 (~70 to 60 ka), and a third interval beginning in late MIS 3 and lasting until MIS 2 (~40 to 18 ka). These intervals of enhanced sediment supply (when compared with today’s values) occur during eustatic sea-level low stands (below −60 to −100 m; Fig. 2*F*) when the PIS most likely covered most of the Patagonian fjords and approached the continental shelf edge. Biogenic sedimentation (low terrigenous input) dominated during interglacial conditions at MIS 5 and the Holocene, together with high eustatic sea-level stands (Fig. 2*F*).

On millennial timescales, all three intervals are characterized by pronounced and reoccurring TIPs lasting between 1.4 and 4.2 ka (Fig. 2 and *SI Appendix*, Table S3). During these millennial TIPs, the distribution of *n*-alkanes indicates a higher proportion of reworked material, consistent with a more important contribution of *n*-alkane deposits associated with glacial erosion of older organic matter sources (**SI Appendix*, Methods*). We labeled all TIPs alphabetically within the associated marine isotope stage and present the different proxy data as mass accumulation rates (MAR) to better document their relative magnitude. The inferred TIPs are shown both in MAR and concentration records in supplemental materials (*SI Appendix*, Fig. S5). In principle, we assume that a greater contribution of terrigenous material indicates increased discharge of the PIS. However, MARs vary among different TIPs and proxies, indicating that the signal might be shaped by local distribution of the different proxies in the source region as well as changing local erosional processes.

A first increased ice discharge of the PIS is recorded in our sediment core for the late MIS 6 around 140 to 130 ka (TIP 6; Fig. 2). During the last glacial–interglacial cycle, initial little changes in sediment supply are already evident in late MIS 5 at ~93.5 and ~88 ka, but too small to be defined as a TIP. The first one (~93.5 ka) shows increased sedimentation rate, titanium accumulation rate, and *n*-alkanes accumulation rates but is not evident in the brGDGTs. The sediment supply at 88 ka is visible in all terrigenous proxies and clearly coincides with an eustatic sea-level low-stand (Fig. 2). Geochronological data of PIS expansion in southeastern Argentina were used to determine that maximum extents were reached earlier at ~93.6 ka and later at ~75 ka [Fig. 2*G* and *SI Appendix*, Table S4; (11, 13)], and underline the possibility of the presence of the PIS on its western central rim. During MIS 4, three events of increased ice discharge, TIP 4a–4c, occurred between ~70 to 65 ka, followed by TIP 4d at ~60 ka. During these three millennial-scale TIPs (4a–4c), MAR proxy records are only marginally lower than during the subsequent MIS 3 TIPs and are clear evidence of a substantially extended PIS at this time (Fig. 2 *D* and *E*). TIP 4d instead, occurs at the very end of MIS 4 with lower MAR values, when eustatic sea level and temperature already increased significantly compared to earlier phases in MIS 4. The occurrence of several TIPs during MIS 4 implies multiple events of increased ice discharge in western–central Patagonia. This time interval is to date not well characterized on land due to subsequent erosion or coverage by later advances during MIS 2 and 3, but larger glacial advances in eastern southern Patagonia at 67.5 and 62.6 ka confirm an extended PIS during this period [Fig. 2*G*; (7, 13, 14, 43)].

During early MIS 3, reduced sedimentation rates of 10 to 15 cm/ka suggest the lack of TIPs, which fits with previous results, showing less favorable conditions for glacier growth at this time (9). The sedimentation rate of 10 to 15 cm/ka is higher than today (~5 cm/ka), and could indicate either a reduced ice discharge of a still extended PIS or increased fluvial sediment transport from the fjord region during the glacial due to a retreated PIS. Based on our multi-proxy sediment core data, it is difficult to distinguish between both scenarios, but the coherence with advances in eastern Patagonia during late MIS 5 (11, 13) lets us suggest that a scenario of a still extended PIS on the continental shelf throughout the entire glacial phase, with varying ice discharge, is most likely.

Two TIPs occurred at ~38 ka (TIP 3a) and ~32 ka (TIP 3b), correlating with major glacier advances at the eastern side of the PIS [Fig. 2*G*; (10, 11, 22)] and suggesting an extended western–central PIS during late MIS 3. During MIS 2, three pronounced TIPs (TIP 2a–c) occurred. The earliest (TIP 2a) developed between ~27 and 25 ka. During this TIP, the terrigenous proxies do not reach the level of the MIS 4 TIPs, except for TIP 4d. In contrast, high MAR of the different proxies characterize TIP 2b and 2c (23 to 20 ka and 20 to 18 ka), suggesting a strongly extended western PIS around the time of the LGM (Fig. 2). The intervals of reduced terrigenous input between the MIS 2 and MIS 3 TIPs denote a substantial decrease of released ice masses into the Pacific at the shelf edge.

After the end of TIP 2c at ~18 ka (Fig. 2), the sedimentation rate at the coring site abruptly dropped from ~80 cm/ka to ~15 cm/ka and continued to decrease over Termination I to Holocene levels of ~5 cm/ka. The timing of this abrupt decrease of sediment supply is in line with previous estimates for the initiation of the deglacial ice sheet retreat in northwestern Patagonia (27, 47, 48). Reconstructed changes of a west-east ice sheet profile at ~47°S also indicate rapid ice sheet thinning starting at ~18 ka and a retreat of the ice sheet to the present location by ~15.5 ka (49). The northeast PIS began to withdraw early, at ~19 ka (50). In contrast, the PATICE study by Davies et al. (4) shows the onset of net ice sheet retreat of the entire PIS as early as 25 ka. Thus, within 5,000 y, the PIS retreated far enough away from the shelf edge to prevent high terrigenous sediment input from reaching our core site. However, Davies et al. (4) note the low confidence in the model retreats due to the lack of well-constrained glaciological records from western Patagonia. Our MIS 2 TIPs between 27 and 18 ka thus provide evidence for an extended PIS during the LGM [defined from 26.5 to 19 ka; (51)] prior to the onset of the last glacial termination. During the Antarctic Cold Reversal [14.6 to 12.8 ka; (52)], the PIS readvanced in eastern southern Patagonia (42), although it had already retreated to a more inland position. In line with these studies, our combined proxies indicate that the PIS indeed did not reach the shelf edge anymore (Fig. 2).

## Changes in Freshwater Input from the Patagonian Ice Sheet

Currently, the Southeast Pacific surface ocean off southern Chile receives substantial amounts of freshwater from the fjord area supplied by rivers, meltwater, and direct rainfall [Fig. 1; (53)]. This freshwater results in a thin layer of low salinity surface waters [<30 m water depth and <33.5 salinity unit; (54)], which is known as Chilean Fjord Water (CFW) flowing northward within ~200 km off the coast [Fig. 1; (54, 55)].

The expansions of the glacial PIS toward the shelf likely produced a substantial meltwater input into the Pacific, resulting in lower surface water salinities off Patagonia. We investigated salinity changes based on two independent proxies: the relative abundance of C_37:4_ alkenones (%C_37:4_; Fig. 3*B*) and δ^18^O data from planktic foraminifera (Fig. 3*C*). C_37:4_ alkenones primarily occur at higher latitudes, where temperatures and salinity are reduced (56). Several studies show a relationship between C_37:4_ alkenones and salinity when C_37:4_ is above 5 % (e.g., refs. 56–58). Our %C_37:4_ record shows substantially elevated values during all TIPs, indicating that the reconstructed ice discharge is connected to lower offshore salinities and temperature minima (*SI Appendix*, *SI Calibration index, and C37:4*). The absolute %C_37:4_ maxima (not dependent on sediment accumulation like the proxy records used to define the TIPs) vary between ~15 and 30%. In contrast to the terrigenous proxy MARs, %C_37:4_ values are overall lower during MIS 4 (10 to 15%) compared to late MIS 3 (20 to 30%). The MIS 2 TIPs 2a–c show values in the range of 15 to 20% (Fig. 3*B*). Intervals of high %C_37:4_ during MIS 2 (but not MIS 3) are also known from the southern core MD07-3128 [*SI Appendix*, Fig. S6*A*; (31)].

The δ^18^O data from planktic, shallow-dwelling (<50 m water depth) foraminifera *Globigerina bulloides* (59) were used to derive qualitative estimates of surface-water paleosalinity changes (Fig. 3*C*). The δ^18^O signal in the foraminiferal tests is a function of local salinity changes as well as changing global ice volume and ambient temperature changes (e.g., ref. 60). The δ^18^O *G. bulloides* record reveals a high short-term variability with amplitudes of ~1.2‰ during the late MIS 3 to MIS 2 TIPs, which cannot be explained by temperature or sea-level changes. During the TIPs, we observe a decrease in δ^18^O, which could be explained by increased freshwater supply and/or warmer ocean temperature. However, our sea surface temperature (SST) reconstructions do not show a distinct warming trend, so increased freshwater supply is most likely contributing to the decline in the δ^18^O signal (Fig. 3*D*).

Similar high amplitudes can also be seen in δ^18^O values from nearby core MD07-3088 (29, 30) between 20 and 18 ka (*SI Appendix*, Fig. S6*B*), which is located ~50 km distance from land (Fig. 1) and thus more proximal to continental freshwater runoff than our study site (~150 km). These δ^18^O records thus provide additional evidence for freshwater input and reduced surface water salinities, largely consistent with our %C_37:4_ records (Fig. 3*B*).

## PIS Variability and Paleoclimate Context over the Past ~140 ka

The timing and paleoclimatic forcing of Quaternary glaciations in the Southern Hemisphere midlatitudes and their links to Northern Hemisphere mountain glaciations and ice sheet development have been discussed for several decades (e.g., refs. 9, 61–63). These studies are primarily based on continental records (e.g., radionuclide-dated moraines) and focus on the last glacial–interglacial cycle.

On orbital timescales, core MR16-09 PC03 provides a continuous marine record documenting an extended PIS during MIS 6, MIS 4, late MIS 3, and MIS 2, i.e., during global glacial maxima with eustatic sea-level low stands (below −60 m) when the PIS covered most of the Chilean fjords and approached the continental shelf edge. Overall, these orbital-scale times of extended ice cover are consistent with reconstructions based on continental records from southern South America and New Zealand (Fig. 2*G*; e.g., refs. 9, 22). Our marine record shows high terrigenous contributions of the PIS already before the global LGM, consistent with most South American glacier chronologies (e.g., refs. 4, 7, 9, and 10). In contrast to previous studies, our record shows a prominent extended PIS during MIS 4 (Fig. 2).

On a global scale, glacier extent in midlatitudes is primarily temperature-controlled (e.g., refs. 64 and 65). Unlike moraine-based PIS reconstructions, our marine proxies allow us to assess SSTs and glacier variations from the same record (Fig. 3*D*). In a maritime setting, SSTs (~150 km off the glacial PIS) plausibly relate to atmospheric temperatures (8, 66). The alkenone-based SST values show strong orbital-scale changes with both U^K’^_37_-based and U^K^_37_-based SSTs (Fig. 3*D* and **SI Appendix*, Methods*). Reconstructed Last Interglacial Maximum (MIS 5.5) SSTs (U^K’^_37_ and U^K^_37_) are ~16 °C, i.e., about 2 °C warmer than during the Holocene. SSTs during glacials MIS 6 and MIS 4–2, on the other hand, were on average ~6° to 9 °C, yielding a glacial–interglacial temperature difference of ~5° to 8 °C. This glacial decrease is consistent with previous alkenone-based SST reconstructions across a larger latitudinal range along the Chilean margin [*SI Appendix*, Fig. S7 and S8; (26, 29, 31)], and corresponds to the predicted cooling from glacier modeling studies required for an expanded PIS (4, 5, 7). The overall coherent chronologies of the New Zealand and South American glaciations during late MIS 3 and MIS 2 suggest common orbital-scale forcing mechanisms resulting in large-scale atmospheric changes in the Southern Hemisphere, such as latitudinal shifts of the SWW belt and the ACC system with its oceanic fronts (6, 7, 9, 47, 61). Thus, the results of our study, showing a re-occurring extended western PIS during MIS 6 and MIS 4, provide critical evidence for the assumption of a general Pacific-wide pattern on orbital timescales beyond the last glacial.

## Millennial-Scale PIS Variations

On millennial timescales, glacial advances in Patagonia and New Zealand have been mostly attributed to ocean cooling in the southern midlatitudes during Antarctic stadials (9, 10, 31, 61). Our phases of increased ice discharge likewise correspond with Antarctic stadials (Fig. 4*B*). This is particularly evident for two stadials that correspond with prominent Dansgaard–Oeschger (DO) warm events in the Northern Hemisphere (e.g., DO8 and DO16; Fig. 4*C*). Some advances during peak glacial intervals MIS 4 and MIS 2 do not match exactly the pronounced Northern Hemisphere interstadials (e.g., TIP 4b, 2a and 2c). During early MIS 3, neither all Antarctic cold phases nor their DO equivalents DO14–9 are significantly represented. An Antarctic millennial-scale timing of oceanic and atmospheric changes in the Southern Hemisphere is consistent with the bipolar seesaw concept (67) of antiphase temperature changes as derived from Greenland and Antarctic ice-core records (e.g., ref. 68). Our millennial-scale SST fluctuations are mostly consistent with other palaeoceanographic records, mainly in the subantarctic realm (26, 27, 69, 70), which document SST cooling during Antarctic stadials (Fig. 4*D*). At the same time, ACC throughflow in the Drake Passage weakened (Fig. 4*E*), and winter sea ice in the Scotia Sea extended equatorward [Fig. 4*F*; (32, 71)], linked with a derived northward shift/expansion of the SWW belt. Such a shift would also plausibly strengthen the northward transport of colder waters from the Southeast Pacific Gyre and ACC to our core position (32) and support an assumed amplified oceanic cooling pattern along the southern Chilean margin. SSTs are generally warmer during early MIS 3 at the northernly sediment core ODP 1233 (~41°S) and decrease continuously between ~60 to 45 ka. A similar trend can be seen between ~60 to 50 ka in the southerly located sediment core MD07-3728 (53°S; *SI Appendix*, Fig. S8). The lack of a prolonged cooling, as in MIS 4 or MIS 2, may have prevented glacier advances (9). Additionally, ACC throughflow in the Drake Passage was enhanced (Fig. 4*E*), and winter sea ice in the Scotia Sea was more limited [Fig. 4*F*; (71)], indicating more poleward aligned SWW/ACC system and accordingly weakened transport of cold water masses to the north. The PIS begins to resemble Antarctic millennial-scale variations again in late MIS 3, although no persistent temperature minima comparable to MIS 4 or MIS 2 occur, indicating that mechanisms other than temperature, such as precipitation, may constitute additional forcing factors.

The amplitude of our U^K’^_37_-derived SST variations during phases of increased ice discharge on millennial timescales is ~2 °C (Fig. 4*D*). The expected SST cooling during TIPs and Antarctic stadials are, however, not consistently observed (Fig. 4 *B* and *D*). While TIPs 3b, and 2a correlate with SST cooling, TIPs 4a-c, and 2b-c are either characterized by no evident SST trend or even show a slight warming. This pattern is difficult to explain since high-resolution SST reconstructions from ODP Site 1233 (~41°S) and MD07-3128 (53°S) document an Antarctic timing of millennial-scale SST variations along the Chilean margin (26, 31). U^K^_37_-derived SST records instead, show a pronounced cooling during phases of increased ice discharge with most amplitudes around 2.5 °C and higher amplitudes of ~5 to 9 °C during TIP 3a and 3b (Fig. 4*D*). It is possible that the alkenone unsaturation indices might be affected by sediment and freshwater outflow from the central PIS, bringing in alkenones from other haptophyte species that differ in their biochemical response to growth temperature from those locally growing haptophytes [*SI Appendix*, *SI Calibration index and C37:4*; (74)]. The more distal and northern site GeoB3327-5 shows %C_37:4_ values <10 [*SI Appendix*, Figs. S5 and S7; (35)], indicating that the high freshwater input does not extend that far offshore. We recommend here a U^K^_37_-based calibration (*SI Appendix*, *SI Calibration index and C37:4*) although the amplitude of SST changes is high.

An important feature of the proxy records indicative of phases of increased ice discharge is their abrupt transitions both at the onset and end of each TIP (Fig. 4*G*). These transitions are also evident in the %C_37:4_ and *G. bulloides* δ^18^O values indicating large and abrupt freshwater inputs associated with Antarctic stadials (Fig. 3*C*). The abrupt character of the signal has mainly two implications. First, it may characterize a PIS threshold with sudden changes in terrigenous sediment supply when the ice sheet reaches the continental shelf and becomes marine-based at its western margin. Second, changes of the western PIS directly exposed to the SWW from the Pacific might be partly precipitation-driven.

It is commonly thought that the coupled SWW/ACC system north of the Southern Ocean fronts might react more abruptly to the bipolar seesaw than Antarctic temperature (e.g., refs. 32, 70, and 75), especially in the southeast Pacific sector. Changes in the strength and position of the SWW likely reinforce glacier advances regionally through enhanced snow accumulation. Western Patagonia is today characterized by a temperate hyper-humid climate (8) with high precipitation (5,000 to 10,000 mm per year) originating from South Pacific moisture transported by the westerlies (4). The positive mass balance would result in PIS expansion toward the outer Pacific shelf edge. This expansion could result in a higher sensitivity of the outer PIS to abrupt temperature changes and thus initiate a higher susceptibility to millennial-scale variations as recorded here in freshwater runoff and linked terrigenous sediment deposition. Such an assumption is in line with the study by Tapia et al. (36) where the high nutrient inflow during MIS 2–4 is considered to be derived from increased precipitation combined with a more active ice sheet. Furthermore, the onset of the TIPs during late MIS 3, when temperature levels were warmer and eustatic sea level higher than during MIS 4 and MIS 2, indicates that increasing precipitation associated with a northward movement of the SWW/ACC system plays an important role. A reason for this northward movement of the SWW/ACC system, as well as the sea ice extent at ~40 ka, could be a decreasing southern hemispheric summer insolation (Fig. 4*A*), which changes the seasonality pattern and thus the position of the SWW/ACC system (61). Overall, it is unclear which mechanisms are directly responsible for the abrupt changes in sediment supply during MIS 6 and the last glacial period, but TIPs occurred when eustatic sea level and temperatures were low and precipitation most likely increased.

Finally, increased orographic precipitation during glacial maxima over the western PIS could cause the eastern part to become substantially drier (13), which would be a possible explanation for some offsets of our results to studies based on the eastern side of the Andes. However, the generally good agreement between the times of western extended ice cover and eastern advances (Fig. 2) suggests synchronous, rather than asynchronous, behavior of ice sheet activity. In addition, uncertainties in our age model and exposure dating make it difficult to accurately compare the timing of western extended ice cover and eastern advances. Nevertheless, in the marine realm, continuous, high-resolution sediment records combined with multiple dating approaches provide a large potential to assign millennial-scale climate patterns unambiguously. Despite remaining age uncertainties, our late MIS 3 and MIS 2 TIPs, can be assigned to Antarctic stadials (i.e., TIP 3a–b and TIP 2a–c; Fig. 4).

## Conclusions

We provide a continuous marine sediment record of the timing and magnitude of marginal fluctuations of the western PIS over a complete glacial–interglacial cycle (~140 ka). Our multiproxy-based study documents major, abrupt changes in ice discharge/meltwater of the central PIS in the southeast Pacific, which occurred during Southern Hemisphere cold phases at MIS 6, MIS 4, late MIS 3, and MIS 2. This record is consistent with detailed, but temporally discontinuous continental ice reconstructions from eastern Patagonia. In addition to Southern Hemisphere temperature control, we suggest that part of the increased ice discharge was also precipitation-driven, as the western PIS might have reacted more sensitively to increases in snowfall. Large amounts of glaciogenic sediments reached the open ocean, explaining the abrupt increase in terrigenous sediment input at our site. Mechanistically, increased ice discharge during Antarctic cooling phases combined with increased precipitation are linked to the northward movement of the coupled subantarctic atmosphere–ocean system.

Our conclusion regarding similarities between PIS activity and Southern Hemisphere climate is consistent with findings of previous continental ice sheet reconstructions. For these continental ice reconstructions, exposure dating has been widely used to attribute individual advances to millennial climate patterns. However, it remains difficult to independently and unambiguously derive the necessary precision in dating for MIS 2, MIS 3, and, to an even larger extent, MIS 4 for continental ice reconstructions. Geological uncertainties in dating moraines and the inherently incomplete nature of the glacial record on land do not allow for precise correlation to individual Southern Hemisphere stadials (13). Herein lies the significance of our sediment record, complementing essential gaps of (western–central) PIS activity. Furthermore, since the location of our site is in an ideal position for studying changes in continental-ocean interactions during the glacial, it presents the potential to reconstruct the PIS activity beyond MIS 6, to times when most of the land-based evidence may have been lost through subsequent glacial erosion.

## Methods

We measured long-chain *n*-alkanes, alkenones, and GDGTs in sediment core MR16-09 PC03. We extracted and separated 224 samples following the method proposed by Auderset et al. (76; **SI Appendix*, Methods*). In short, the sediment was simultaneously extracted and separated into two compound classes using an accelerated solvent extractor (ASE). The first fraction (long-chain *n*-alkanes and alkenones) was analyzed using a gas chromatograph with a flame ionization detector (GC-FID) 7890BGC System from Agilent Technologies. The second fraction GDGT was analyzed in a High-Performance Liquid Chromatograph (HPLC) coupled to a single quadrupole mass spectrometer detector (Agilent Technologies). Furthermore, we used an ITRAX micro-XRF scanner to determine titanium. A Thermo MAT253 mass spectrometer and a Thermo MAT253Plus were used for determining the stable oxygen isotope ratio (δ^18^O) of planktic foraminifera (*G. bulloides* and *G. truncatulinoides*). Values are reported as ‰ vs. V-PDB. The age model is based primarily on tuning δ^18^O of *G. truncatulinoides* to the δ^18^O intermediate Pacific stack of Lisiecki and Stern (39) on orbital timescales and on ^14^C dating (*G. bulloides*) on millennial timescales. For the radiocarbon dating, we calibrated our samples using MARINE20 (77) and an ΔR of 400 y (78). Further age tie points were provided by magnetostratigraphic data, documenting the Laschamps geomagnetic excursion (41 ka), embedded in a relative paleointensity minimum. A detailed description of the *Methods* can be found in *SI Appendix*.

## Supplementary Material

Appendix 01 (PDF)

## Data Availability

Excel file containing data collection of the piston core MR16-09 PC03 in the southeast Pacific data have been deposited in PANGAEA (79).
