# Supplementary material for: A marine record of Patagonian ice sheet changes over the past 140,000 years
Source: Proc Natl Acad Sci U S A. 2024 Mar 4;121(12):e2302983121. doi: 10.1073/pnas.2302983121 (PMC10962970; doi:10.1073/pnas.2302983121)
Supplement: Supplementary file 1 — Appendix 01 (PDF) [file pnas.2302983121.sapp.pdf]

Supporting Information for

A marine record of Patagonian ice sheet changes over the past 140,000 years.

Julia R. Hagemann[a,b,1], Frank Lamy[a,c,1], Helge W. Arz[d], Lester Lembke-Jene[a], Alexandra Auderset[b,e], Naomi Harada[f,g], Sze Ling Ho[h], Shinya Iwasaki[i], Jérôme Kaiser[d], Carina B. Lange[j,k,l], Masafumi Murayama[m,n], Kana Nagashima[g], Norbert Nowaczyk[o], Alfredo Martínez-García[b] & Ralf Tiedemann[a,c]

[a]Division of Geoscience, Marine Geology Section, Alfred Wegener Institute Helmholtz Centre for Polar and Marine Research, 27570 Bremerhaven, Germany; [b]Department of Climate Geochemistry, Organic Isotope Geochemistry Group, Max Planck Institute for Chemistry, 55128 Mainz, Germany; [c]Center for Marine Environmental Sciences, University of Bremen, 28359 Bremen, Germany; [d]Department of Marine Geology, Paleoceanography and Sedimentology Group, Leibniz Institute for Baltic Sea Research Warnemünde, 18119 Rostock, Germany; [e]School of Ocean and Earth Science, University of Southampton SO17 1BJ, Southampton, United Kingdom; [f]Atmosphere and Ocean Research Institute, Center for International and Local Research Cooperation, The University of Tokyo, 277-

8564 Kashiwa, Japan; [g]Research Institute for Global Change, Earth Surface System Research Center, Japan Agency for Marine-Earth Science and Technology, 237-0061 Yokosuka, Japan; [h]Institute of Oceanography, National Taiwan University, 10617 Taipei, Taiwan; [i]Graduate School of Environmental Science, Hokkaido University, 060-0810 Sapporo, Japan; [j]Departamento de Oceanografía & Centro de Investigación Oceanográfica en el Pacífico Suroriental (Coastal), Universidad de Concepción, 4030000 Concepción, Chile; [k]Centro de Investigación Dinámica de Ecosistemas Marinos de Altas Latitudes (IDEAL), Universidad Austral de Chile, 5110566 Valdivia, Chile; [l]Scripps Institution of Oceanography, University of California San Diego, La Jolla, CA 92037, United States; [m]Faculty of Agriculture and Marine Science, Kochi University, Nankoku, 783-8502 Kochi, Japan; [n]Center for Advanced Marine Core Research, Kochi University, Nankoku, 783-8502 Kochi, Japan; [o]Department of Geosystems, Section of Climate Dynamics and Landscape Evolution, Helmholtz Centre Potsdam German Research Centre for Geosciences, 14473 Potsdam, Germany

[1]Julia R. Hagemann and Frank Lamy.

Email: Julia.Hagemann@awi.de; Frank.Lamy@awi.de

This PDF file includes:

Supporting text

Figures S1 to S9

Tables S1 to S4

SI References

SI Age Model

At orbital timescales, we fitted the $\delta^{18}O$ record of the intermediate-dwelling foraminifera *Globorotalia truncatulinoides* to the $\delta^{18}O$ intermediate Pacific stack (IP; **Fig**. S2, **Table** S1; 1). We preferred planktic deep dwellers, which reflect intermediate water mass characteristics (2, 3) over benthic foraminifers because the latter might be influenced by high, but largely unconstrained, ages of Pacific Deep Water masses prevalent at the study site (4). In particular, during glacials and glacial terminations, aged Pacific Deep Water (5, 6) may well lead to unanticipated temporal offsets when aligned with reference stack records, which may not fully compensate for such bias (1, 7, 8). In contrast, intermediate waters in the study area are not known to exhibit similar aging behavior (9) and were thus used here. Based on the good agreement between the EDML (10) oxygen isotope reference record and the XRF-based Rubidium (Rb) record from our site, thought to reflect fine-grained terrigenous input as a clay mineral component, we added four more tuning points, mainly around MIS 4 (**Fig**. S2), which are all within the principal error ranges of the existing oxygen isotope-based age model. We checked our age model for consistency with $\delta^{18}O$ of *G. bulloides* and alkenone-derived SSTs on EDML, but did carry not out further adjustments.

The age model of the younger section of our core (<42 ka) is based on ^{14}C dating (*G. bulloides*). We calibrated our samples with MARINE20 (11) and an ΔR of 400 years (12) using the program Calib 8.2 (**Table** S2). We choose all dating points between the TIPs (**Fig**. S2) to avoid the major impact of glacial meltwater. We also associated all TIPs with Antarctic stadials, considering the 2-sigma range of the radiocarbon calibration-derived

calendar ages. An association of all TIPs with Antarctic interstadials instead was not possible for TIPs 2c, 2b, and 3b. In addition, we also used the onset and termination of the geomagnetic Laschamps excursion centered at 41 ka, which matches well with two ^{14}C ages (**Fig**. S3; **Table** S2). We conclude that the millennial-scale variations during MIS 3 and MIS 2 are related to Antarctic timing, indicating PIS advances occurring during stadials.

SI Methods

Sediment Core. Piston core MR16-09 PC03 was retrieved from the Southeast Pacific, ~150 km west of the Chilean coast (46° 24.32' S, 77° 19.45' W; 3082 m water depth) during RV Mirai Cruise Leg 2 of the Trans South Pacific Project Expedition (13). The 17.53 m long sediment core consists mainly of glacial gray to olive gray silty clay with diatoms and nannofossils. Lighter, more carbonate-rich intervals are restricted to the thin Holocene sequence at the top and the last interglacial sediments towards the bottom of the core, accompanied by increased biogenic components. In addition, a ~15 cm thick dark brownish tephra layer in core depth ~9.1 m was identified as a turbidite and removed from the core sequence.

Biomarkers. A total of 224 samples, taken every 4.3 cm in the interglacial intervals and 8.6 cm in the glacial intervals, were analyzed for *n*-alkanes, alkenones, and isoprenoid (not used in this study), and branched glycerol dialkyl glycerol tetraethers (GDGTs). Sample

preparation, extraction, and measurement were done at the Max Planck Institute for Chemistry in Mainz, according to the method described in Auderset *et al.* (14). In this method, the biomarker extraction was done by an Accelerated Solvent Extraction (DIONEX ASE 350; Thermo Scientific). During the extraction, the samples were separated into two fractions (fraction I containing *n*-alkanes and alkenones; fraction II containing GDGTs) through using an ASE. The ASE cells were filled with 16 g silica gel (deactivated by adding 5 % milli-Q water) and 2 g of freeze-dried and homogenized sediment samples. Subsequently, the cells were filled with solvent and heated up to 100° C for 5 min. The extraction was performed using *n*-hexane (15 ml; 1-time), DCM (22 ml; 3-times), and DCM:MeOH (1:1, v/v; 22 ml; 3-times). Fractions I (*n*-Hexane and DCM) and II (DCM:MeOH) were collected separately. After ASE extraction, 80 µl of 2-nonadecanone and hexatriacontane were added to fraction I and 60 µl of C_{46}-GDGT (15, supplied by Pandion Labs) to fraction II as internal standards. In a Rocket Evaporator (Genevac – SP Scientific), both fractions were dried under vacuum and converted into 1.5 ml vials. For the measurement, fraction I was dissolved in 30 – 80 µl isooctane, and 5 µl were injected into a GC-FID, a Gas Chromatography – Flame Ionization Detector (GC-FID) 7890B GC System from Agilent Technologies with helium as the carrier gas, a VF200ms column (60 m x 250 µm x 0.25 µm), and a PTV injector. The oven temperature increased from 44 to 300° C at 20° C/ min and held at 300° C for 14 minutes. Fraction II was diluted in 400 µl *n*-hexane:isopropanol (1.5 %) and 20 µl were injected in a HPLC-MS, a High-Performance Liquid Chromatography (Agilent, 1260 Infinity) with a single quadrupole mass spectrometer detector (Ag-

ilent 6130) with the method as described in Hopmans *et al.* (16). We measured the following masses: *m/z* 744 (C_{46} standard), *m/z* 1302.3 (GDGT-0), *m/z* 1300.3 (GDGT-1), *m/z* 1298.3 (GDGT-2), *m/z* 1296.3 (GDGT-3), *m/z* 1292.3 (crenarchaeol and crenarchaeol isomer), *m/z* 1022 (GDGT-Ia), *m/z* 1020 (GDGT-Ib), *m/z* 1018 (GDGT-Ic), *m/z* 1036 (GDGT-IIa and IIa'), *m/z* 1034 (GDGT-IIb and IIb'), *m/z* 1032 (GDGT-IIc and IIc'), *m/z* 1050 (GDGT-IIIa and IIIa'), *m/z* 1048 (GDGT-IIIb and IIIb'), *m/z* 1046 (GDGT-IIIc and IIIc'). We used the GC Analysis Software ChemStation by Agilent Technologies to quantify *n*-alkanes and alkenones and Agilent MassHunter Quantitative Analysis Version B.07.01 for isoprenoid- and branched GDGTs.

Mass Accumulation Rate (MAR). *n*-alkanes, brGDGT and titanium (Ti), considered here as terrestrial proxies (e.g., 17, 18-22), are presented as mass accumulation rates (MAR). We determined the accumulation rate as follows (23, 24):

$$MAR = SR * terrigenous\ Input * DBD$$

MAR – Mass Accumulation Rate [ng/ka*cm^2], SR - Sedimentation rate [cm/ka], DBD - Dry Bulk Density [g/cm^3], and terrigenous input based on the concentrations of *n*-alkanes and branched GDGTs [ng/g Sed.]. For Ti, counts per second (cps) were used to determine MAR instead of concentrations. Concentrations, counts per second, and MAR of the *n*-alkanes, brGDGTs, and Ti can be found in **Fig**. S5.

***n*-alkanes and branched GDGTs.** Long-chain (C_{27} – C_{33}) *n*-alkanes are leaf waxes from terrestrial plants that can be used to infer changes in the transport of terrestrial material to the ocean (17). We used the carbon preference index (CPI), which compares even and odd carbon numbered *n*-alkanes, to asses potential changes in *n*-alkanes sources through time (**Fig**. S5*B*). CPI values of 1 are typical of mature samples, while leaf waxes are characterized by high CPI values (25, 26). In the absence of petrogenic markers, low CPI values have been attributed to increased microbial reworking of the odd carbon numbered *n*-alkanes (22, 27). At our site the CPI varies between ~5-6 during warm phases and ~3 during millennial scale cold periods when the terrestrial input is the highest. These observations suggest a change in *n*-alkane sources during these events. The higher CPI values observed during the warmer intervals point towards a dominant contribution from unaltered higher plant material that may have been transported to our core site by runoff or winds. In contrast, the higher proportion of microbially altered *n*-alkanes during these events is consistent with a more important contribution of older *n*-alkane deposits transported to the sediment by glacial erosion associated with the advance of the PIS.

The average chain length (ACL; **Fig**. S5*C*), which traces changes in the distribution of odd *n*-alkanes, can provide information on the source vegetation, with higher values (i.e. 30.66±0.83) typically associated with C4 grasses and lower values (i.e. 29.00±0.83) associated with C3 trees, shrubs and grasses (26). At our site ACL values range between ~30 during warmer intervals and 29.7 during millennial-scale cold periods. These observations are consistent with wetter conditions during these periods favoring the development of a larger proportion of C3 vegetation.

Branched GDGTs (brGDGTs) are produced mostly in soils (28). The branched vs isoprenoid index (BIT; **Fig**. S5*E*) provides information about the terrigenous fraction of all GDGTs in the sediment, with higher values indicating a dominance of soil GDGTs (19). However, it has also been shown that in some open ocean location the BIT can affected by the production and/or preferential degradation of isoprenoid GDGTs, and high BIT values can be obtained despite of lower brGDGTs inputs (20). Thus, it is recommended to compare BIT values with brGDGTs concentrations and MARs (20). Our BIT index shows higher values during the TIPs, together with higher concentrations and MAR of brGDGTs indicating a higher proportion of soil-derived organic matter during these events.

Sea surface temperature (SST). We used the Unsaturation Ketone index (U^{K}_{37} – index; U^{K}_{37} = [$C_{37:2}$]-[$C_{37:4}$]/[$C_{37:2}$]+[$C_{37:2}$]+[$C_{37:4}$]) with a chain length of 37 carbon atoms, with the di-, tri- and tetra-unsaturated alkenones $C_{37:2}$, $C_{37:3}$ and $C_{37:4}$ (29) and the modified $U^{K'}_{37}$ – index ($U^{K'}_{37}$ = [$C_{37:2}$] /[$C_{37:2}$]+[$C_{37:2}$]) after Prahl and Wakeham (30) excluding the tetra-unsaturated alkenone $C_{37:4}$. The SSTs based on both indices were calculated with SST = (U^{K}_{37} + 0.104)/0.04 (31) and SST' = ($U^{K'}_{37}$ - 0.044)/0.033 (32).

XRF-Scan. Scanning X-ray fluorescence spectroscopy was performed by an ITRAX micro-XRF scanner at Kochi Core Center, Kochi University, Japan, using a Mo X-ray tube with settings of 30 kV, 55 mA, down-core step sizes of 0.5 cm and a counting time of 15 seconds per step.

δ^{18}O. The stable oxygen isotope ratio (δ^{18}O) of planktic foraminifera were measured in the Marine Geology stable isotope laboratory facility at the Alfred-Wegener-Institute in Bremerhaven, Germany. 121 samples of *G. bulloides* (size range 250–500 μm), in addition to the dataset of Iwasaki *et al.* (4), and a total of 171 samples of *G. truncatulinoides* (size range 250–500 μm) were picked to determine δ^{18}O. Analyses were carried out on a Thermo MAT253 mass spectrometer and a Thermo MAT253Plus, both connected to Kiel IV CARBO units. All values are reported as ‰ vs. V-PDB. Calibration was obtained with NIST 19 and an internal carbonate standard of Solnhofen limestone.

Magnetostratigraphy. The core was sampled every ~23 mm with cubic plastic boxes (6 cm^3). Remanence Measurements were performed with a 2G Enterprises superconducting long-core cryogenic magnetometer with in-line 3-axis alternating field demagnetizer. All samples were stepwise demagnetized and demagnetization results were subjected to principal component analysis in order to determine the characteristic remanent magnetization (ChRM). An anhysteretic remanent magnetization (ARM) was imparted as a proxy for the concentration of magnetic particles and subsequently also stepwise demagnetized. The slope of NRM versus ARM of common demagnetization levels was then used to determine the relative paleointensity (RPI). Documented geomagnetic excursion (Laschamps 41 ka), as well as the obtained RPI curve, correlated to the reference curve provided by Liu *et al.* (33), provided age tie points.

SI Calibration index and $C_{37:4}$

The two indices, U^{K}_{37} and $U^{K'}_{37}$, differ in alkenone $C_{37:4}$, which occurs mainly in cold regions (e.g., 34) and was therefore removed from the global $U^{K'}_{37}$ index (30). The choice of the index at this site is critical for a qualitative statement about the effect of temperature on ice sheet dynamics, as they are inconsistent during TIPs. The alkenone $C_{37:4}$ is particularly conspicuous because it is present in relatively large amounts (10 – 25 %) during TIPs (**Fig**. S9*A* – gray area). Otherwise, %$C_{37:4}$ is ≤10, with only minor differences between the indexes (**Fig**. S9*A*). This large amount of %$C_{37:4}$ during TIPs is reflected in a linear relationship (**Fig**. S9*B*) between U^{K}_{37} and $U^{K'}_{37}$ (when slope = 1, then $U^{K}_{37} = U^{K'}_{37}$), with a slope of ~0.9 (%$C_{37:4}$ < 5), ~0.8 (%$C_{37:4}$ between 5 – 10), and ~0.2 (%$C_{37:4}$ > 10). Studies have demonstrated a correlation of $C_{37:4}$ with the temperature only when %$C_{37:4}$ ≤ 5 (35-37), implying that the U^{K}_{37} – based temperature calibration at our site is biased during TIPs.

In addition to cold temperatures, $C_{37:4}$ also appears to correlate with salinity negatively (34, 36, 37). The correlation between increasing %$C_{37:4}$ and decreasing salinity could be due to (1) an adjustment in the biosynthesis of the ocean-dominant haptophyte *Emiliania huxleyi* or (2) increased occurrence of other alkenone-forming haptophyte species. However, adjustment of *E. huxleyi* biosynthesis results in only a small increase (≤10 %$C_{37:4}$) (35), suggesting that salinity changes during TIPs are not the only reason for the high %$C_{37:4}$ values at our site. High (>10) %$C_{37:4}$ values could be attributed in some studies to other alkenone-forming haptophyte species that exhibit a different biochemical response to growth temperature than *E. huxleyi* (e.g., 35, 38). *E. huxleyi* and *Gephyrocapsa oceanica* are the predominant alkenone-producing species in the ocean (e.g., 39, 40, 41) but may be

locally complemented by other haptophyte species, e.g., in regions of sea ice formation (42). They also occur in considerable genetic variation in lakes, where they have higher levels of unsaturation (e.g., 38).

High %$C_{37:4}$ values were also observed in previous studies. For example, Bendle *et al.* (34) found high values (40 – 77 %) of %$C_{37:4}$ in polar waters, intermediate values (0 – 25 %) in Arctic and Norwegian coastal waters, and low values (0 – 3 %) in Atlantic waters. This high occurrence of %$C_{37:4}$ in polar and coastal waters supports the assumption that other haptophyte species may have influenced sites in regions with sea ice or increased terrigenous input. %$C_{37:4}$ data from core GeoB3327-5 (**Fig**. S9*C*; 43), located ~425 km further offshore, shows no %$C_{37:4}$ values >10 for ~500 ka, implying that freshwater input never reaches this site. Other %$C_{37:4}$ offshore data from site PS75/034-2 (**Fig**. S9*D*; 43) further south at 54° S instead show $C_{37:4}$ values up to 20 % during glacial phases. In this case, the elevated %$C_{37:4}$ values could be related to northward extending sea ice during the glacials (44, 45). The sea ice most likely did not reach the site but the increased northward expansion of Antarctic water masses (46, 47) may have transported alkenones from the phylogenetic Group of 2i Isochrysidales which are associated with sea ice (42) to site PS75/034-2.

The high %$C_{37:4}$ values during TIPs at our site suggest that alkenones deposited in the sediment are not exclusively from *E. huxleyi*. Other alkenone-forming haptophyte species could have been deposited autochthonous as well as allochthonous. The sharp drop in $\delta^{18}O$ of the surface-dwelling foraminifer *Globigerina bulloides* during the TIPs supports a large freshwater influx that may have caused a shift in the haptophyte community. On the other

hand, the high %$C_{37:4}$ values are consistent with a high terrigenous input (e.g., *n*-alkanes, Ti, brGDGTs), which does not exclude alkenones produced in lakes. Furthermore, the differential biochemical response of other haptophyte species to growth temperature most likely also affects the $U^{K'}_{37}$ – index. Therefore, Alkenone SSTs during TIPs cannot be unambiguously determined at our site due to the potential influence of other haptophyte species. Instead, we use here %$C_{37:4}$ as an additional proxy for freshwater supply. The terrigenous influence during the TIPs on the SSTs is illustrated by the absence of the characteristic orbital-scale fluctuations, like MIS 4 or the last glacial maximum (LGM), which are not detectable in $U^{K'}_{37}$ – derived SSTs and only hinted at in U^{K}_{37} – derived SSTs. Studies north at ~41° S (**Fig**. S8*B*; 48, 49) and south at ~53° S (**Fig**. S8*E*; 50) of our site show a very prominent MIS 4 and LGM, instead.

In summary, both indices are most likely influenced by other haptophyte species at our site. However, the U^{K}_{37} – index shows a consistent pattern of cooler SSTs during TIPs, broadly consistent with Antarctic stadials and coolings in the more northern site ODP 1233 and southern MD07-3128. Haptophytes living in lakes have a higher degree of unsaturation but still, show a correlation with temperature, so U^{K}_{37} – based calibrations are applied (38). With such an influence from freshwater-induced haptophyte community or lake-derived alkenones, $C_{37:4}$ would still correlate with temperature, albeit in a different biogeochemical dependence. Such a correlation of the alkenone $C_{37:4}$ with temperature is consistent with studies from the North Atlantic and Nordic Seas with a high number of samples north of 70° N, an area of sea ice formation, showing a stronger correlation of the U^{K}_{37} – index than the $U^{K'}_{37}$ – index with SST (51, 52) and no correlation of the $U^{K'}_{37}$ – index with SST at all

(36). It is possible, although highly speculative, that U^{K}_{37} – index is also the better choice at our site and reflects the general temperature changes, albeit with a larger amplitude than expected.

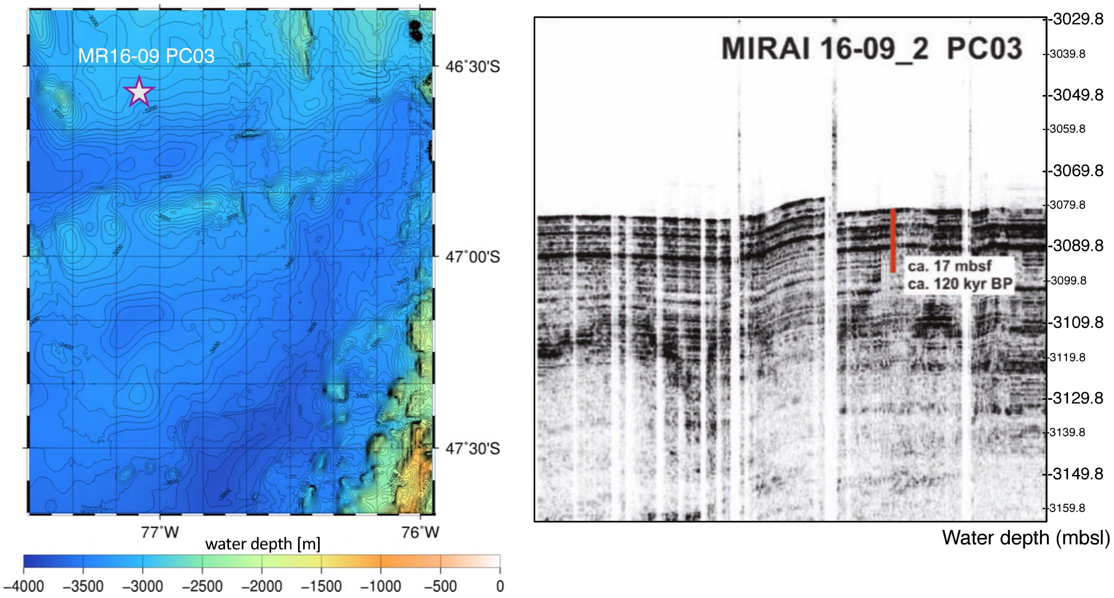

Fig. S1. Site location of MR16-09 PC03 with a bathymetric map (left) and 3.5 kHz acoustic sub-bottom profiler (right).

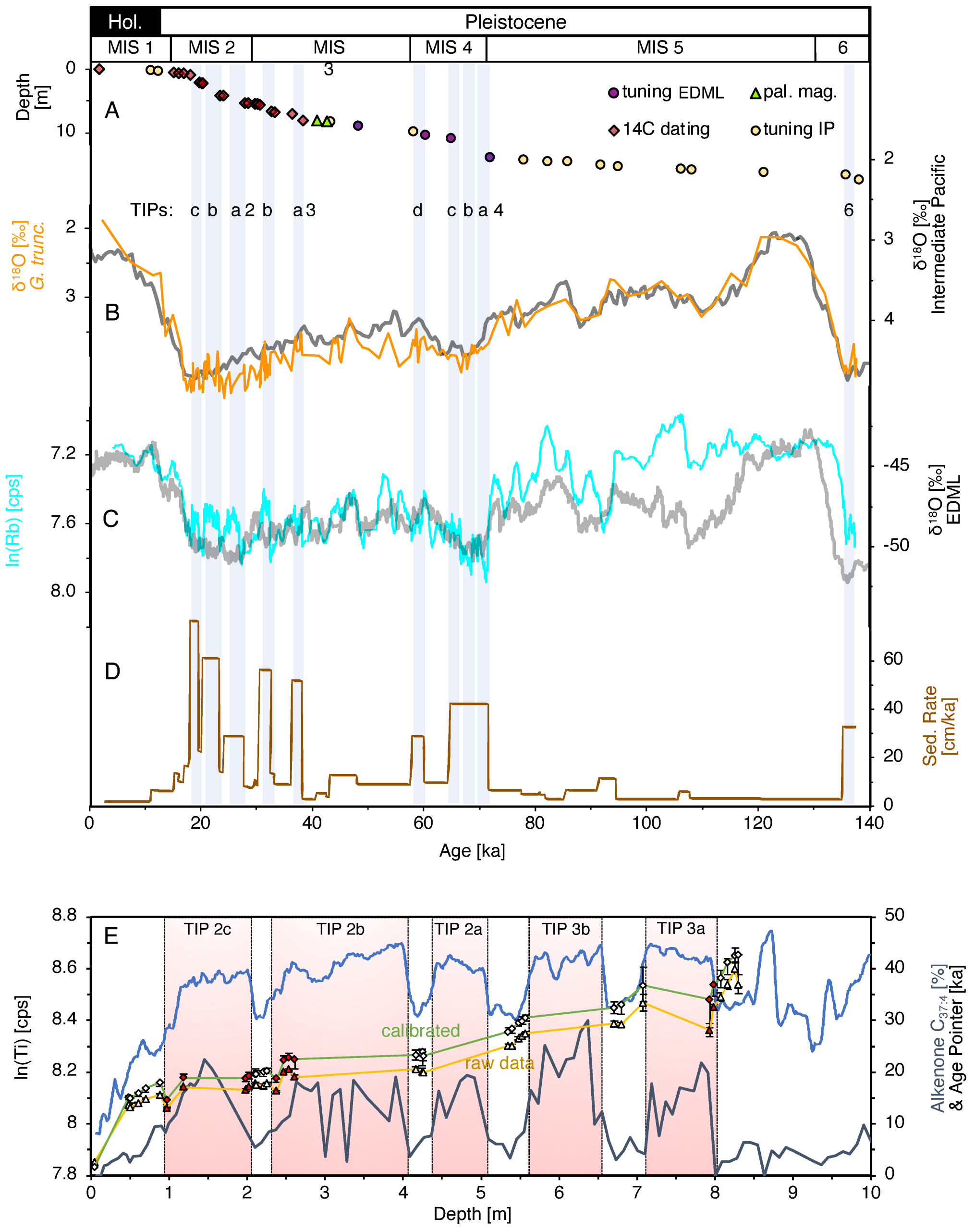

Fig. S2. Age model of core MR16-09 PC03. Gray shadings and numbers at the top mark the times of the Terrigenous Input Phase (TIP). (**A**) ^{14}C and tuning points. (**B**) $\delta^{18}O$ values of Intermediate Pacific waters (IP; 1) with $\delta^{18}O$ values of the deep-dwelling (200 - 500 m; 53, 54) foraminifer *G. truncatulinoides*. (**C**) $\delta^{18}O$ values of the Antarctic ice core EDML

(10) with XRF-scan of the element composition of rubidium (Rb). (**D**) Sedimentation rate of the Age model. (**E**) Depth vs. age plot of surface-dwelling foraminifer *Globigerina bulloides* ^{14}C raw data (yellow) with the error of measurement and ^{14}C calibrated data (green) with MARINE20 (11), an ΔR of 400 years (12), and an error bar showing the 2-sigma range. In the background are shown XRF-derived ln(Ti) and the %$C_{37:4}$ alkenone to evaluate the sections of terrigenous input. Only samples from sections with little terrigenous input were chosen for the age model to avoid a bias of glacier meltwater. Red-shaded stipes show sections of high terrigenous input. The age model did not consider red-marked ^{14}C age pointers (raw and calibrated data).

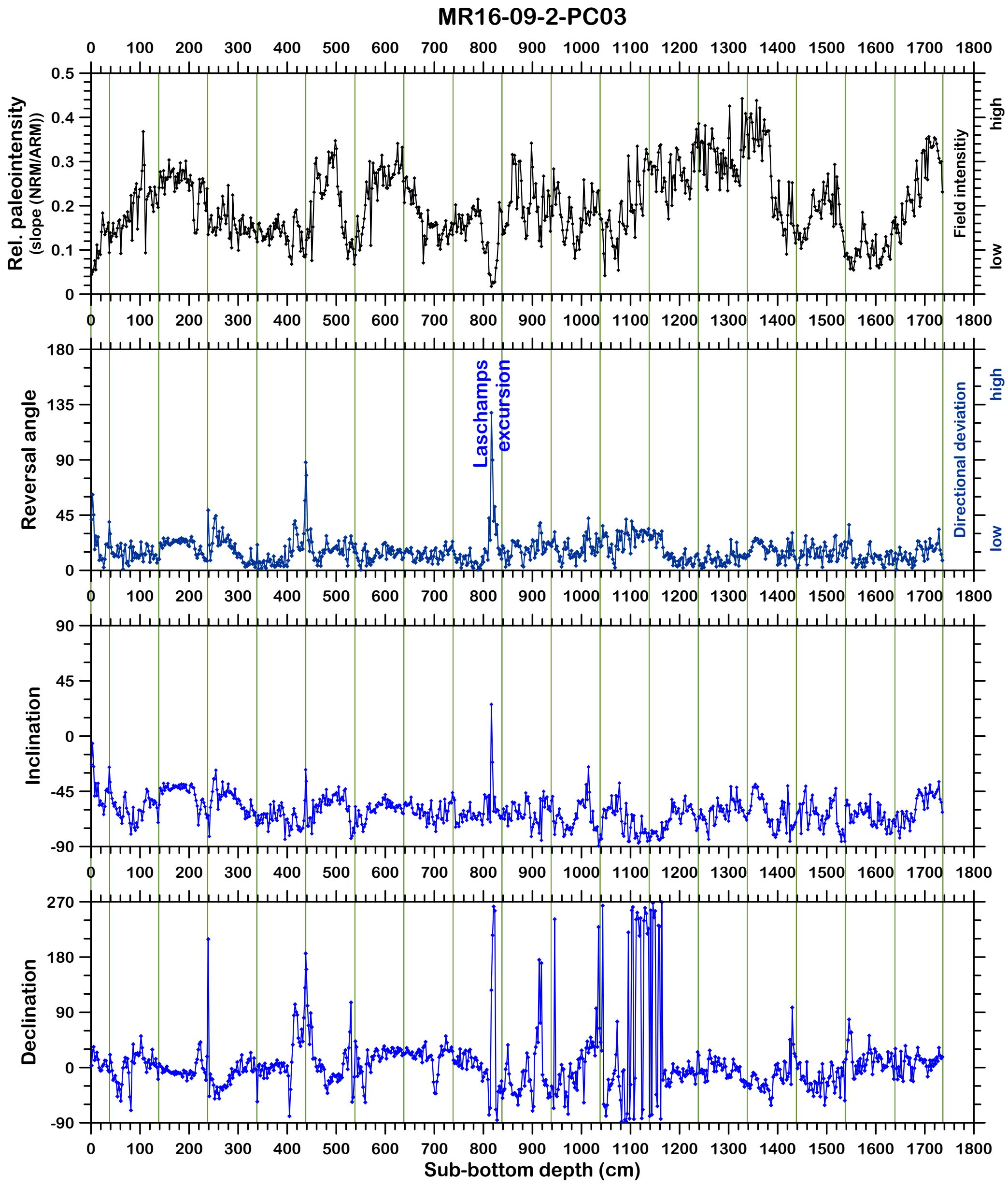

Fig. S3. Magnetostratigraphic results from Mirai core PC03: (**a**) relative paleointensity estimated by the slope of NRM (natural remanent magnetization) versus ARM (anhysteretic remanent magnetization) of common demagnetization steps, (**b**) reversal angle, defined as the angle along a great circle between the expected dipole direction and the measured di-

rection, shown as Inclination in (**c**) and declination in (**d**). Directions pointing up and northward (down and southward) are associated with normal (reversed) polarity. The geomagnetic Laschamps excursion at 41 ka (55) is also associated with low (relative) paleointensities.

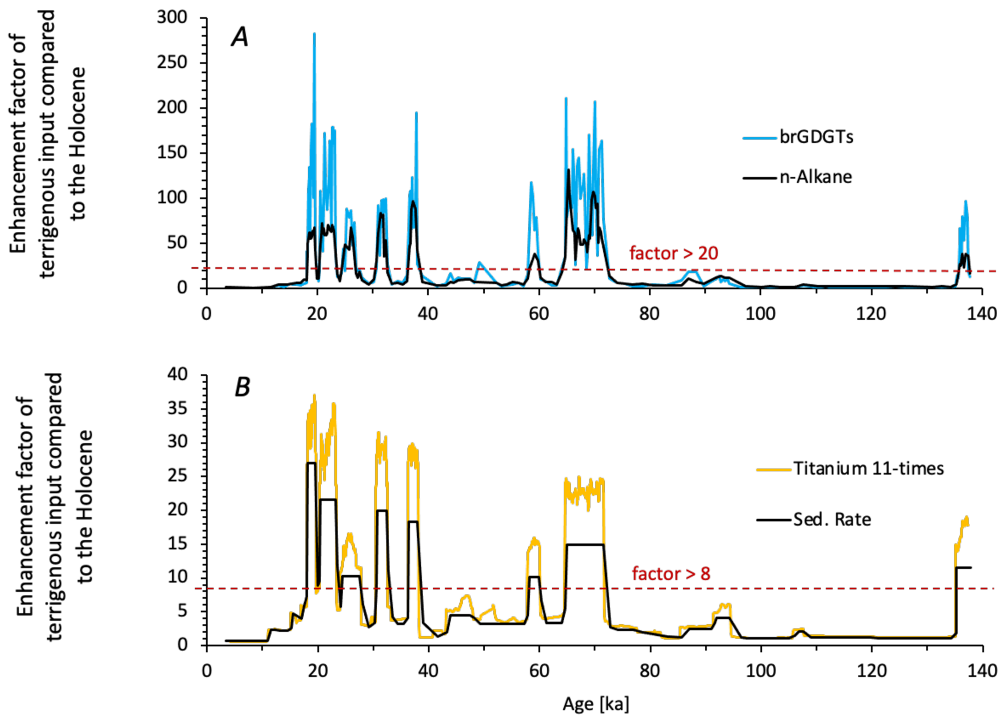

Fig. S4. Biomarker (A) and sedimentation rate (B) normalized to the averaged Holocene background sedimentation.

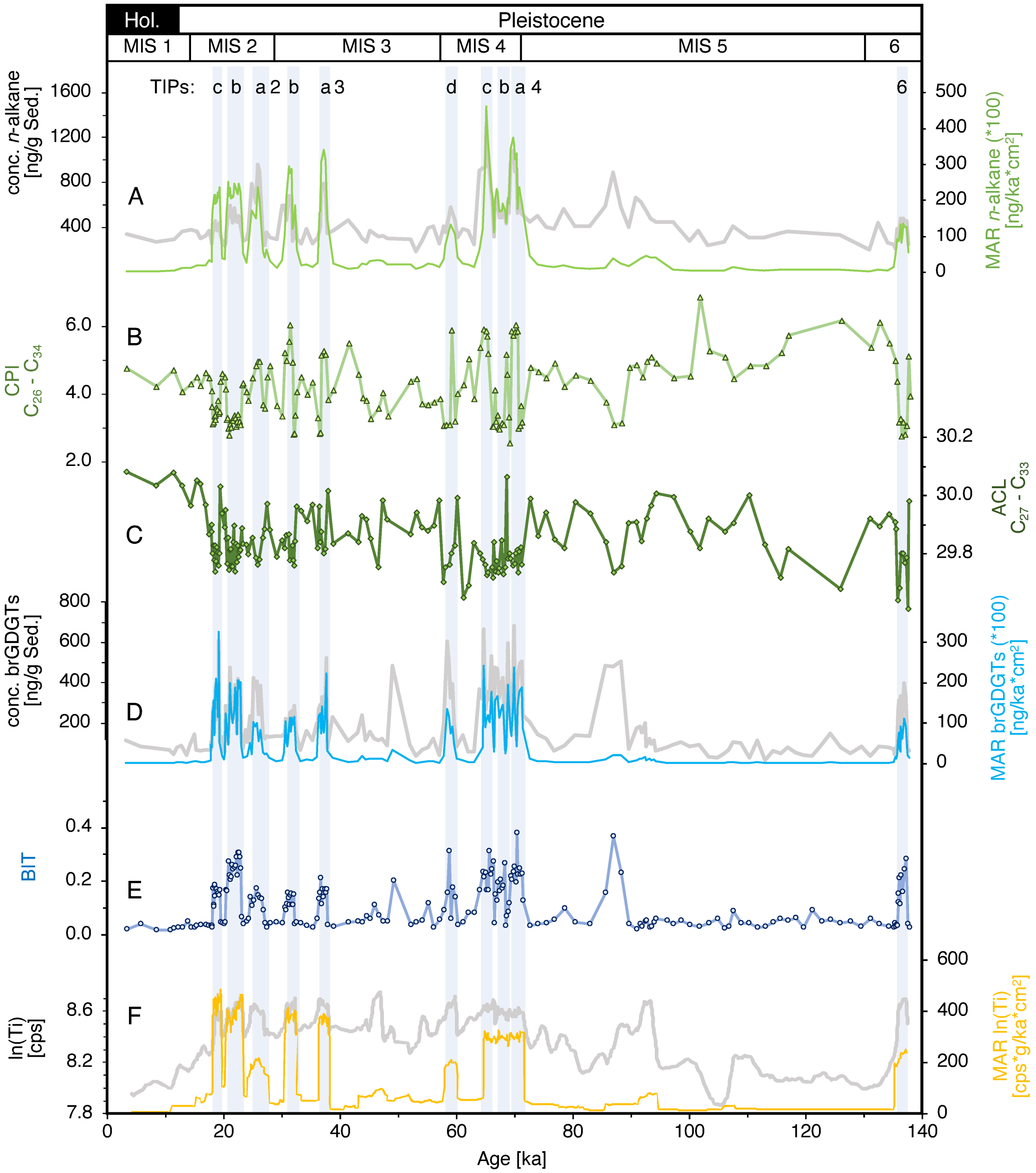

Fig. S5. Concentrations (left axis) and Mass Accumulation Rates (MAR; right axis) of *n*-alkanes (**A**). Carbon preference index (CPI) for the *n*-alkanes $C_{26} - C_{34}$ (**B**). Average chain length (ACL) for the *n*-alkanes $C_{27} - C_{33}$ (**C**). Concentrations (left axis) and Mass Accu-

mulation Rates (MAR; right axis) of branched GDGTs (brGDGTs) (**D**). Branched vs isoprenoid index (BIT) to determine the terrigenous fraction in the sediment (**E**). Concentrations (left axis) and Mass Accumulation Rates (MAR; right axis) of Titanium (ln(Ti)), whereby the MAR of Ti is based on their cps and not on their concentration. Furthermore, for Ti, an 11-point running mean was chosen (**F**). Gray shadings and numbers at the top mark Terrigenous Input Phases (TIP).

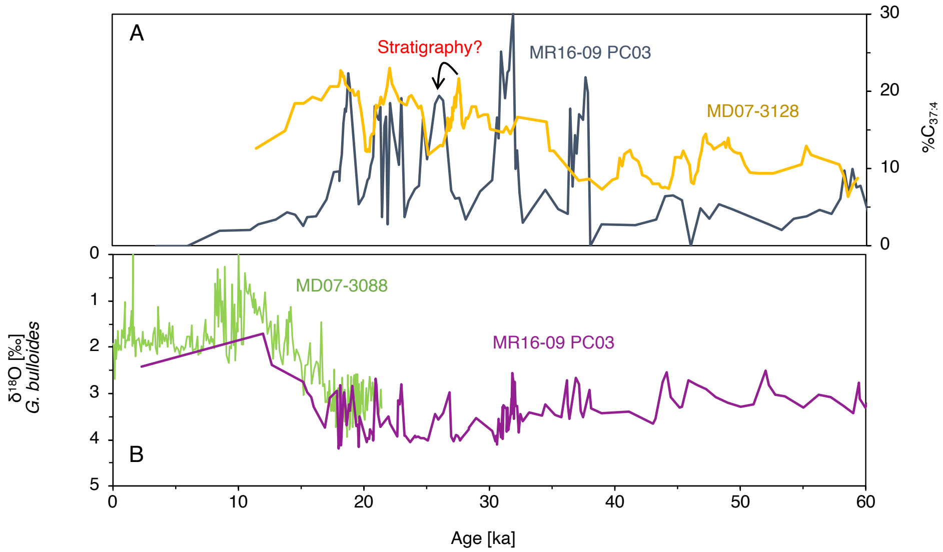

Fig. S6. Comparison of the alkenone %$C_{37:4}$ of the southern core MD07-3128 (yellow line; 50) with MR16-09 PC03 (gray line; this study). The data of core MD07-3128 were cleaned from outliers and shown here as a 5-point moving average (**A**). Comparison of the $\delta^{18}O$ values of the foraminifer *G. bulloides* of the nearby core MD07-3088 (green line; 56, 57) with MR16-09 PC03 (violet line; this study) (**B**).

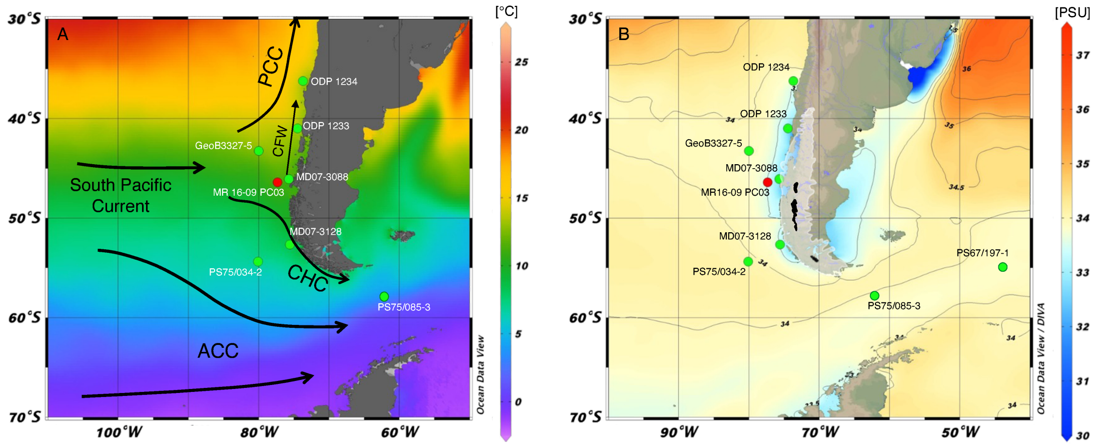

Fig. S7. World Ocean Atlas (WOA13) derived (**A**) sea surface temperature (SST; 58) and (**B**) sea surface salinity (SSS; 59) maps of the study area with for this paper relevant cores sites (GeoB3327-5, PS75/034-2: 43, PS75/085-3, PS67/197-1 47, ODP 1233: 48, MD07-3128: 50, MD07-3088: 56, ODP 1234: 60). ACC: Antarctic Circumpolar Current; PCC: Peru-Chile Current; CHC: Cape Horn Current; CFW: Chilean Fjord Water; Semi-transparent pale shading: Patagonian Icesheet (PIS) during its maximum extension. Black polygons: Recent icefields in Patagonia (61).

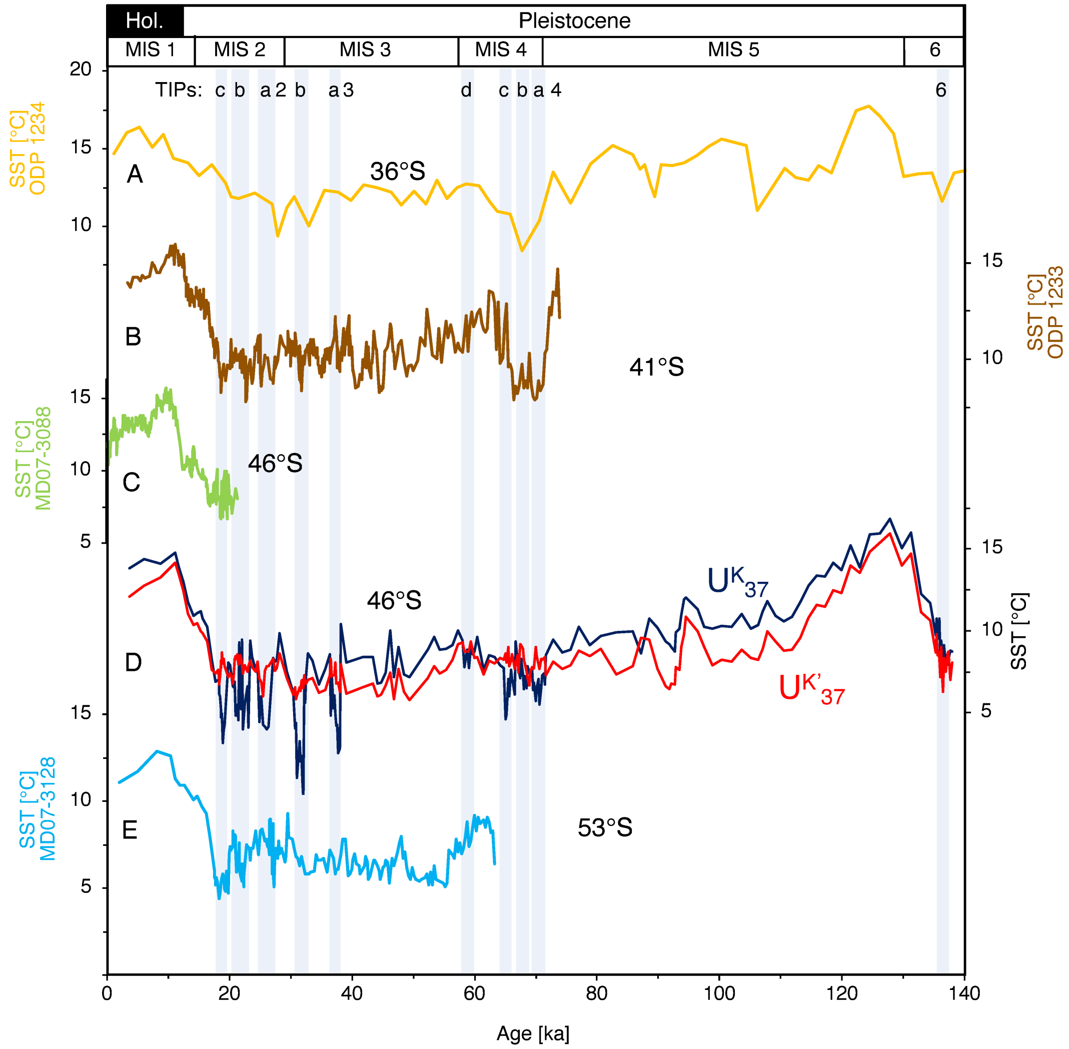

Fig. S8. SSTs ($U^{K'}_{37}$) of additional cores along the Chilean continental margin from north to south. Gray shadings and numbers at the top mark Terrigenous Input Phases (TIP). (**A**) ODP site 1234 (60). (**B**) ODP Site 1233 (48, 49). (**C**) MD07-3088 (56, 57). (**D**) MR16-09 PC03 (this study). (**E**) MD07-3128 (50).

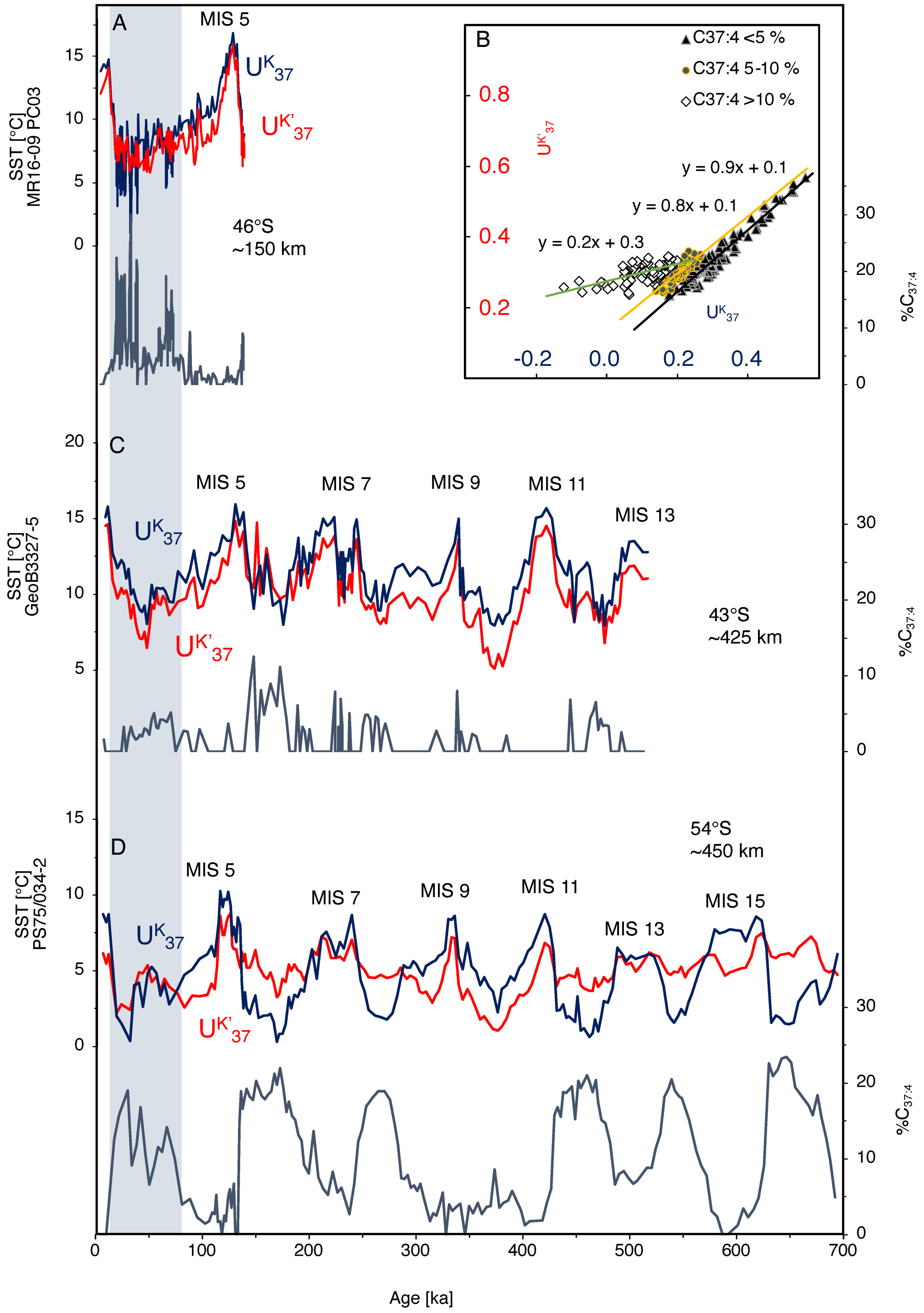

A
MIS 5
SST [°C] MR16-09 PC03
46°S
~150 km
B
C37:4 <5 %
C37:4 5-10 %
C37:4 >10 %
y = 0.9x + 0.1
y = 0.8x + 0.1
y = 0.2x + 0.3
C
SST [°C] GeoB3327-5
MIS 5
MIS 7
MIS 9
MIS 11
MIS 13
43°S
~425 km
D
SST [°C] PS75/034-2
54°S
~450 km
MIS 5
MIS 7
MIS 9
MIS 11
MIS 13
MIS 15
Age [ka]

Fig. S9. U^{K}_{37} (gray; 31), $U^{K'}_{37}$ (red; 32) based SSTs and $\%C_{37:4}$ of cores (A) MR16-09 PC03 (this study), (C) GeoB3327-5 and (D) PS75/034-2 from Ho *et al.* (43; $\%C_{37:4}$ published in this study). Gray marked area shows the last glacial with high $\%C_{37:4}$ values of this study. (B) Relationship of U^{K}_{37} – index, and $U^{K'}_{37}$ – index of core MR16-09 PC03.

Table S1. Age pointers of the age model.

Depth [m]	Age [ka]			Depth [m]	Age [ka]	
0.04	1.69	^{14}C dating		8.14	40.68	Laschamps
0.21	10.9	$\delta^{18}O$ tuning		8.23	42.47	Laschamps
0.3	12.28	$\delta^{18}O$ tuning		8.26	43.06	$\delta^{18}O$ tuning
0.48	15.08	^{14}C dating		8.88	47.97	$\delta^{18}O$ tuning
0.61	16	^{14}C dating		9.78	57.79	$\delta^{18}O$ tuning
0.7	16.9	^{14}C dating		10.43	60.05	$\delta^{18}O$ tuning
0.88	18	^{14}C dating		10.87	64.55	$\delta^{18}O$ tuning
2.11	19.6	^{14}C dating		13.84	71.59	$\delta^{18}O$ tuning
2.12	19.65	^{14}C dating		14.22	77.5	$\delta^{18}O$ tuning
2.21	20.05	^{14}C dating		14.43	81.79	$\delta^{18}O$ tuning
2.26	20.24	^{14}C dating		14.53	85.34	$\delta^{18}O$ tuning
4.16	23.37	^{14}C dating		14.93	91.31	$\delta^{18}O$ tuning
4.25	24	^{14}C dating		15.29	94.41	$\delta^{18}O$ tuning
5.35	27.80	^{14}C dating		15.61	105.71	$\delta^{18}O$ tuning
5.40	28.43	^{14}C dating		15.73	107.6	$\delta^{18}O$ tuning
5.48	29.52	^{14}C dating		16.16	120.43	$\delta^{18}O$ tuning
5.53	29.96	^{14}C dating		16.58	135.09	$\delta^{18}O$ tuning
5.57	30.47	^{14}C dating		17.36	137.48	$\delta^{18}O$ tuning
6.71	32.49	^{14}C dating				
6.80	33.17	^{14}C dating				
7.08	36.2	^{14}C dating				
8.07	38.12	^{14}C dating				

Table S2. All ^{14}C data based on *G. bulloides* for the age model of core MR16-09 PC03. The calibration is based on MARINE20 (11) with a reservoir age of 400 years (12) in the Program Calib. 8.2. Samples were excluded if they were located directly in a TIP event; shaded samples were considered in the age model. Two (1875, 2209; red) samples could not be included in the age model, even considering the maximum 2-sigma range and under the assumptions described in the text above. A third sample (7373) was removed because it did not fit the following $\delta^{18}O$ tuning. Samples 8315 and 8318 corresponded to the onset and termination of the paleomagnetic marker Laschamps.

Internal AWI Lab-Nr.	Core Depth [cm]	^{14}C raw Age [ka]	± Error Age [ka]	1-Sigma Age [ka]	1-Sigma Age [ka]	2-Sigma Age [ka]	2-Sigma Age [ka]	Used Age [ka]
**2206.1.1**	4.3	2.657	0.074	1.572	1.795	1.469	1.915	1.686
**2207.1.1**	48.4	13.632	0.123	14.886	15.27	14.631	15.488	15.08
**1875.1.1**	50.6	13.251	0.140	14.216	14.758	14.004	14.972	
**7360.1.1**	60.8	14.104	0.143	15.486	15.923	15.265	16.122	16
**7361.1.1**	70	14.819	0.152	16.403	16.849	16.184	17.036	16.9
**7363.1.1**	88.4	15.674	0.135	17.452	17.876	17.277	18.079	18
**7364.1.1**	97.2	13.051	0.126	13.882	14.344	13.739	14.664	
**1876.1.2**	119.2	17.142	0.132	19.102	19.485	18.899	19.681	
**8333.1.1**	197.9	16.518	0.165	18.379	18.798	18.197	18.987	
**8320.1.1**	202.6	17.144	0.286	18.927	19.636	18.665	20.04	
**1877.1.1**	210.8	17.821	0.167	19.907	20.362	19.634	20.564	19.6
**7365.1.1**	212	17.594	0.156	19.616	20.072	19.427	20.298	19.65
**8313.1.1**	221.1	17.462	0.180	19.448	19.943	19.196	20.195	20.05
**8332.1.1**	225.5	17.915	0.179	20.006	20.482	19.764	20.739	20.244
**1878.1.1**	238	16.303	0.153	18.216	18.586	18.025	18.755	
**7366.1.1**	247.7	20.099	0.184	22.552	22.995	22.345	23.24	
**2208.1.1**	253.4	20.490	0.191	22.975	23.472	22.819	23.726	
**8334.1.1**	261.4	19.019	0.385	21.115	22.071	20.613	22.456	
**8331.1.1**	416.1	20.613	0.411	22.911	23.804	22.419	24.278	23.369
**8327.1.1**	425	20.127	0.674	22.152	23.653	21.228	24.385	24
**2209.1.1**	426.1	19.987	0.185	22.466	22.891	22.248	23.094	
**7367.1.1**	534.6	25.067	0.265	27.694	28.29	27.443	28.604	27.795

**7368.1.1**	539.6	25.093	0.270	27.713	28.318	27.461	28.635	28.426
**7369.1.1**	547.9	26.559	0.305	29.213	29.843	28.887	30.102	29.524
**7370.1.1**	552.6	26.989	0.315	29.62	30.301	29.238	30.646	29.957
**7371.1.1**	557.1	27.530	0.320	30.167	30.785	29.912	31.033	30.472
**8314.1.1**	670.8	29.465	0.453	31.865	33.068	31.382	33.644	32.49
**8306.1.1**	679.9	29.227	0.376	31.689	32.729	31.254	33.168	33.168
**8317.1.1**	707.6	33.409	1.625	35.204	38.759	33.526	40.329	36.866
**8325.1.1**	793.5	28.128	1.299	29.81	32.644	28.552	34.027	
**8307.1.1**	798	32.528	0.514	35.207	36.266	34.634	36.843	
**8308.1.1**	806.9	34.555	0.746	37.293	39.085	36.34	39.679	38.234
**8315.1.1**	816.3	36.914	0.924	39.727	41.214	38.934	41.896	41.21
**8318.1.1**	825.6	40.054	1.041	41.835	43.089	41.246	44.017	42.489
**7373.1.1**	830.2	37.009	1.869	38.904	41.99	36.429	42.747	

Table S3. Terrigenous Input Phases (TIP). The age was read from the terrigenous entries, at the beginning and end of each TIP. DO: Dansgaard-Oeschger

TIP	Depth [m]		Age [ka]		Duration [ka]	Tentative to DO
6	17.38	16.79	137.5	135.7	1.8	-
4a	13.91	12.55	72.7	68.5	4.2	DO18?
4b	12.55	1.73	68.5	66.6	1.9	-
4c	11.73	10.73	66.6	63.1	3.5	DO17?
4d	10.45	9.73	60.3	57.2	3.1	**DO16**
3a	8	7.1	38.0	36.2	1.8	**DO8**
3b	6.64	5.55	32.4	30.2	2.2	DO5?
2a	5.19	4.27	27.3	24.1	3.2	-
2b	4.09	2.37	23.3	20.4	2.8	DO2?
2c	2.1	1	19.6	18.2	1.4	-

Table S4. Selection of previously published land-based advances mainly from Patagonian glaciers of moraines and out-washed sediments and used for Figures 2 and Figure 4. [1]Radiocarbon dating, recalibrated with SHCal13 by Davies *et al.* (61; file "Radiocarbon_ages", tab "Terrestrial", column "W"). The difference to the originally used IntCal13 calibration of Moreno *et al.* (62) are negligible. We used an arithmetic average calculation and the standard error (1σ) of several dating points for each advancing event. [2]Cosmogenic dating, recalibrated with Lm scaling and an erosion rate of 0 mm/ka by Davies *et al.* (61; file "^{10}Ba and ^{26}Al ages August 2019", tab "0 erosion Kaplan et al." column "Y"). We used an arithmetic average calculation and the standard error (1σ) of several dating's for each advancing event. For single advancing events, we used the external standard deviation (given in column "AA"). [3]Data, based on Lm scaling, originally taken from the reference.

**Reference**	**Advances (ka)**						
**North Patagonia: 38 – 46° S, west side of the Andes**							
[1]Moreno *et al.* (62)	17.8 ± 0.6	25.8 ± 1.4	26.7 ± 0.3	30.9 ± 0.8	33.3 ± 0.5		
[3]García *et al.* (63)	26.0 ± 2.9	57.8 ± 4.7					
**Central Patagonia: 46 – 50° S, east side of the Andes**							
[2]Hein *et al.* (64)	17.4 ± 1.7	19.2 ± 1.8	20.9 ± 1.2	24.9 ± 0.5	28.4 ±1.0		
[3]Mendelova *et al.* (65)	24.8 ± 1.0	75.2 ± 2.8					
[2]Glasser *et al.* (66)	15.3 ± 1.7	23.5 ± 2.2	35.2 ± 3.2	(60.0 ± 5.9)	93.6 ± 3.8		
**South Patagonia: 50 – 53° S, east side of the Andes**							
[2]García *et al.* (67), (68)	14.1 ± 0.5	21.7 ± 2.0	35.5 ± 4.1	40.0 ± 4.1	48.3 ± 1.8		
[2]Kaplan *et al.* (69)	20.1 ± 0.9	21.4 ± 2.1	23.3 ± 1.3	28.7 ± 1.2			
[3]Peltier *et al.* (70)	18.1 ± 0.6	19.1 ± 0.7	23.9 ± 0.8	25.7 ± 0.8	27.4 ± 0.8	65.4 ± 2.0	67.5 ± 2.1
**New Zealand**							
[3]Strand *et al.* (71)	18.0 ± 0.4	20.0 ± 0.5	26.7 ± 0.7	36.5 ± 0.9	41.8 ± 1.1	44.0 ± 1.0	
[3]Schaefer *et al.* (72)	65.1 ± 2.7						

SI References

1. L. E. Lisiecki, J. V. Stern, Regional and global benthic δ18O stacks for the last glacial cycle. *Paleoceanography* **31**, 1368-1394 (2016).
2. R. Tapia *et al.*, Glacial differences of Southern Ocean Intermediate Waters in the Central South Pacific. *Quaternary Science Reviews* **208**, 105-117 (2019).
3. R. Tapia, D. Nurnberg, T. Ronge, R. Tiedemann, Disparities in glacial advection of Southern Ocean Intermediate Water to the South Pacific Gyre. *Earth and Planetary Science Letters* **410**, 152-164 (2015).
4. S. Iwasaki *et al.*, Evidence for late-glacial oceanic carbon redistribution and discharge from the Pacific Southern Ocean. *Nat Commun* **13** (2022).
5. T. A. Ronge *et al.*, Radiocarbon constraints on the extent and evolution of the South Pacific glacial carbon pool. *Nat Commun* **7**, 11487 (2016).
6. T. A. Ronge, M. Sarnthein, J. Roberts, F. Lamy, R. Tiedemann, East Pacific Rise Core PS75/059-2: Glacial-to-Deglacial Stratigraphy Revisited. *Paleoceanography and Paleoclimatology* **34**, 432-435 (2019).
7. L. Lin, D. Khider, L. E. Lisiecki, C. E. Lawrence, Probabilistic sequence alignment of stratigraphic records. *Paleoceanography* **29**, 976-989 (2014).
8. L. E. Lisiecki, M. E. Raymo, Diachronous benthic δ18O responses during late Pleistocene terminations. *Paleoceanography* **24** (2009).
9. R. De Pol-Holz, L. Keigwin, J. Southon, D. Hebbeln, M. Mohtadi, No signature of abyssal carbon in intermediate waters off Chile during deglaciation. *Nature Geoscience* **3**, 192-195 (2010).
10. E. C. Members, One-to-one coupling of glacial climate variability in Greenland and Antarctica. *Nature* **444**, 195-198 (2006).
11. T. J. Heaton *et al.*, Marine20-the Marine Radiocarbon Age Calibration Curve (0-55,000 Cal Bp). *Radiocarbon* **62**, 779-820 (2020).
12. T. Heaton *et al.*, 10.31223/x5p92g (2022).
13. JAMSTEC (2018) Trans South Pacific Project. in *MIRAI MR16-09 Leg2 Cruise Data*.

14. A. Auderset, M. Schmitt, A. Martinez-Garcia, Simultaneous extraction and chromatographic separation of n-alkanes and alkenones from glycerol dialkyl glycerol tetraethers via selective Accelerated Solvent Extraction. *Organic Geochemistry* **143** (2020).
15. A. P. Patwardhan, D. H. Thompson, Efficient synthesis of 40- and 48-membered tetraether macrocyclic bisphosphocholines. *Org Lett* **1**, 241-243 (1999).
16. E. C. Hopmans, S. Schouten, J. S. S. Damste, The effect of improved chromatography on GDGT-based palaeoproxies. *Organic Geochemistry* **93**, 1-6 (2016).
17. T. I. Eglinton, G. Eglinton, Molecular proxies for paleoclimatology. *Earth and Planetary Science Letters* **275**, 1-16 (2008).
18. A. Martínez-Garcia *et al.*, Links between iron supply, marine productivity, sea surface temperature, and CO2 over the last 1.1 Ma. *Paleoceanography* **24**, (2009).
19. E. C. Hopmans *et al.*, A novel proxy for terrestrial organic matter in sediments based on branched and isoprenoid tetraether lipids. *Earth and Planetary Science Letters* **224**, 107-116 (2004).
20. S. Fietz *et al.*, Crenarchaea and phytoplankton coupling in sedimentary archives: Common trigger or metabolic dependence? *Limnology and Oceanography* **56**, 1907-1916 (2011).
21. A. Martinez-Garcia *et al.*, Southern Ocean dust-climate coupling over the past four million years. *Nature* **476**, 312-315 (2011).
22. J. Villanueva, J. O. Grimalt, E. Cortijo, L. Vidal, L. Labeyrie, A biomarker approach to the organic matter deposited in the North Atlantic during the last climatic cycle. *Geochimica Et Cosmochimica Acta* **61**, 4633-4646 (1997).
23. P. Blum, *Physical Properties Handbook: A Guide to the Shipboard Measurement of Physical Properties of Deep-Sea Cores*, Ocean Drilling Program Technical Notes (1997), 10.2973/odp.tn.26.1997.
24. T. H. Van Andel, G. R. Heath, T. C. Moore, "Cenozoic History and Paleoceanography of the Central Equatorial Pacific Ocean: a regional synthesis of

Deep Sea Drilling Project data" in Cenozoic History and Paleoceanography of the Central Equatorial Pacific Ocean. (1975), 10.1130/MEM143-p1, pp. 1-223.

25. E. E. Bray, E. D. Evans, Distribution of n-paraffins as a clue to recognition of source beds. *Geochimica et Cosmochimica Acta* **22**, 2-15 (1961).

26. F. Rommerskirchen, A. Plader, G. Eglinton, Y. Chikaraishi, J. Rullkötter, Chemotaxonomic significance of distribution and stable carbon isotopic composition of long-chain alkanes and alkan-1-ols in C4 grass waxes. *Organic Geochemistry* **37**, 1303-1332 (2006).

27. G. Poynter J, Aeolian-derived higher plant lipids in the marine sedimentary record : links with paleoclimate. *Paleoclimatology and Paleometeorology : Modern and Past Patterns of Global Atmospheric Transport*, 435-462 (1989).

28. S. Schouten, E. C. Hopmans, J. S. S. Damste, The organic geochemistry of glycerol dialkyl glycerol tetraether lipids: A review. *Organic Geochemistry* **54**, 19-61 (2013).

29. S. C. Brassell, G. Eglinton, I. T. Marlowe, U. Pflaumann, M. Sarnthein, Molecular Stratigraphy - a New Tool for Climatic Assessment. *Nature* **320**, 129-133 (1986).

30. F. G. Prahl, S. G. Wakeham, Calibration of unsaturation patterns in long-chain ketone compositions for palaeotemperature assessment. *Nature* **330**, 367-369 (1987).

31. F. G. Prahl, L. A. Muehlhausen, D. L. Zahnle, Further Evaluation of Long-Chain Alkenones as Indicators of Paleoceanographic Conditions. *Geochimica Et Cosmochimica Acta* **52**, 2303-2310 (1988).

32. P. J. Müller, G. Kirst, G. Ruhland, I. von Storch, A. Rosell-Melé, Calibration of the alkenone paleotemperature index U37K′ based on core-tops from the eastern South Atlantic and the global ocean (60°N-60°S). *Geochimica et Cosmochimica Acta* **62**, 1757-1772 (1998).

33. J. Liu, N. R. Nowaczyk, S. Panovska, M. Korte, H. W. Arz, The Norwegian-Greenland Sea, the Laschamps, and the Mono Lake Excursions Recorded in a Black Sea Sedimentary Sequence Spanning From 68.9 to 14.5 ka. *Journal of Geophysical Research: Solid Earth* **125** (2020).

34. J. Bendle, A. Rosell-Mele, P. Ziveri, Variability of unusual distributions of alkenones in the surface waters of the Nordic seas. *Paleoceanography* **20** (2005).
35. T. Blanz, K. C. Emeis, H. Siegel, Controls on alkenone unsaturation ratios along the salinity gradient between the open ocean and the Baltic Sea. *Geochimica Et Cosmochimica Acta* **69**, 3589-3600 (2005).
36. A. Rosell-Melé, Interhemispheric appraisal of the value of alkenone indices as temperature and salinity proxies in high-latitude locations. *Paleoceanography* **13**, 694-703 (1998).
37. A. Rosell-Melé, E. Jansen, M. Weinelt, Appraisal of a molecular approach to infer variations in surface ocean freshwater inputs into the North Atlantic during the last glacial. *Global and Planetary Change* **34**, 143-152 (2002).
38. W. J. D'Andrea, S. Theroux, R. S. Bradley, X. Huang, Does phylogeny control U37K-temperature sensitivity? Implications for lacustrine alkenone paleothermometry. *Geochimica et Cosmochimica Acta* **175**, 168-180 (2016).
39. T. D. Herbert, "Alkenone Paleotemperature Determinations" in Treatise on Geochemistry. (2003), 10.1016/b0-08-043751-6/06115-6, pp. 391-432.
40. J. K. Volkman, S. M. Barrett, S. I. Blackburn, E. L. Sikes, Alkenones in Gephyrocapsa-Oceanica - Implications for Studies of Paleoclimate. *Geochimica Et Cosmochimica Acta* **59**, 513-520 (1995).
41. J. K. Volkman, G. Eglinton, E. D. S. Corner, J. R. Sargent, Novel unsaturated straight-chain C37-C39 methyl and ethyl ketones in marine sediments and a coccolithophore Emiliania huxleyi. *Physics and Chemistry of the Earth* **12**, 219-227 (1980).
42. S.-m. Wang, A.-h. Xie, J.-p. Zhu, Does polar amplification exist in Antarctic surface during the recent four decades? *Journal of Mountain Science* **18**, 2626-2634 (2021).
43. S. L. Ho *et al.*, Sea surface temperature variability in the Pacific sector of the Southern Ocean over the past 700 kyr. *Paleoceanography* **27** (2012).

44. V. Benz, O. Esper, R. Gersonde, F. Lamy, R. Tiedemann, Last Glacial Maximum sea surface temperature and sea-ice extent in the Pacific sector of the Southern Ocean. *Quaternary Science Reviews* **146**, 216-237 (2016).
45. R. Gersonde, X. Crosta, A. Abelmann, L. Armand, Sea-surface temperature and sea ice distribution of the Southern Ocean at the EPILOG Last Glacial Maximum - A circum-Antarctic view based on siliceous microfossil records. *Quaternary Science Reviews* **24**, 869-896 (2005).
46. F. Lamy *et al.*, Glacial reduction and millennial-scale variations in Drake Passage throughflow. *Proc Natl Acad Sci U S A* **112**, 13496-13501 (2015).
47. S. Wu *et al.*, Orbital- and millennial-scale Antarctic Circumpolar Current variability in Drake Passage over the past 140,000 years. *Nat Commun* **12** (2021).
48. J. Kaiser, F. Lamy, D. Hebbeln, A 70-kyr sea surface temperature record off southern Chile (Ocean Drilling Program Site 1233). *Paleoceanography* **20** (2005).
49. F. Lamy *et al.*, Antarctic timing of surface water changes off Chile and Patagonian ice sheet response. *Science* **304**, 1959-1962 (2004).
50. M. Caniupán *et al.*, Millennial-scale sea surface temperature and Patagonian Ice Sheet changes off southernmost Chile (53°S) over the past ∼60 kyr. *Paleoceanography* **26** (2011).
51. J. Bendle, A. Rosell-Melé, Distributions of UK37 and UK37′ in the surface waters and sediments of the Nordic Seas: Implications for paleoceanography. *Geochemistry, Geophysics, Geosystems* **5** (2004).
52. A. Filippova, M. Kienast, M. Frank, R. R. Schneider, Alkenone paleothermometry in the North Atlantic: A review and synthesis of surface sediment data and calibrations. *Geochemistry Geophysics Geosystems* **17**, 1370-1382 (2016).
53. I. Wilke, H. Meggers, T. Bickertc, Depth habitats and seasonal distributions of recent planktic foraminifers in the Canary Islands region (29 degrees N) based on oxygen isotopes. *Deep-Sea Research Part I-Oceanographic Research Papers* **56**, 89-106 (2009).

54. A. N. LeGrande, J. Lynch-Stieglitz, E. C. Farmer, Oxygen isotopic composition of Globorotalia truncatulinoidesas a proxy for intermediate depth density. *Paleoceanography* **19** (2004).
55. N. R. Nowaczyk, H. W. Arz, U. Frank, J. Kind, B. Plessen, Dynamics of the Laschamp geomagnetic excursion from Black Sea sediments. *Earth and Planetary Science Letters* **351**, 54-69 (2012).
56. N. A. Haddam *et al.*, Changes in latitudinal sea surface temperature gradients along the Southern Chilean margin since the last glacial. *Quaternary Science Reviews* **194**, 62-76 (2018).
57. G. Siani *et al.*, Carbon isotope records reveal precise timing of enhanced Southern Ocean upwelling during the last deglaciation. *Nat Commun* **4**, 2758 (2013).
58. R. A. Locarnini *et al.*, World Ocean Atlas, 2013, Volume 1: Temperature. S. Levitus, Ed. A. MIshonov, Technical Ed.; NOAA Atlas NESDIS 73. http://www.nodc.noaa.gov/.
59. M. M. Zweng *et al.*, World Ocean Atlas 2013, Volume 2: Salinity. S. Levitus, Ed.; A. Mishonov, Technical Ed.; NOAA Atlas NESDIS 74.
60. M. W. de Bar, D. J. Stolwijk, J. F. McManus, J. S. Sinninghe Damsté, S. Schouten, A Late Quaternary climate record based on long-chain diol proxies from the Chilean margin. *Climate of the Past* **14**, 1783-1803 (2018).
61. B. J. Davies *et al.*, The evolution of the Patagonian Ice Sheet from 35 ka to the present day (PATICE). *Earth-Science Reviews* **204** (2020).
62. P. I. Moreno *et al.*, Radiocarbon chronology of the last glacial maximum and its termination in northwestern Patagonia. *Quaternary Science Reviews* **122**, 233-249 (2015).
63. J.-L. García *et al.*, A composite 10Be, IR-50 and 14C chronology of the pre-Last Glacial Maximum (LGM) full ice extent of the western Patagonian Ice Sheet on the Isla de Chiloé, south Chile (42° S). *Quaternary Science Journal* **70**, 105-128 (2021).
64. A. S. Hein *et al.*, The chronology of the Last Glacial Maximum and deglacial events in central Argentine Patagonia. *Quaternary Science Reviews* **29**, 1212-1227 (2010).

65. M. Mendelova, A. S. Hein, A. Rodes, S. Xu, Extensive mountain glaciation in central Patagonia during Marine Isotope Stage 5. *Quaternary Science Reviews* **227** (2020).
66. N. F. Glasser *et al.*, Cosmogenic nuclide exposure ages for moraines in the Lago San Martin Valley, Argentina. *Quaternary Research* **75**, 636-646 (2011).
67. J.-L. García *et al.*, The MIS 3 maximum of the Torres del Paine and Última Esperanza ice lobes in Patagonia and the pacing of southern mountain glaciation. *Quaternary Science Reviews* **185**, 9-26 (2018).
68. J. L. García *et al.*, Glacier expansion in southern Patagonia throughout the Antarctic cold reversal. *Geology* **40**, 859-862 (2012).
69. M. R. Kaplan *et al.*, Southern Patagonian glacial chronology for the Last Glacial period and implications for Southern Ocean climate. *Quaternary Science Reviews* **27**, 284-294 (2008).
70. C. Peltier *et al.*, The large MIS 4 and long MIS 2 glacier maxima on the southern tip of South America. *Quaternary Science Reviews* **262** (2021).
71. P. D. Strand *et al.*, Millennial-scale pulsebeat of glaciation in the Southern Alps of New Zealand. *Quaternary Science Reviews* **220**, 165-177 (2019).
72. J. M. Schaefer *et al.*, The Southern Glacial Maximum 65,000 years ago and its Unfinished Termination. *Quaternary Science Reviews* **114**, 52-60 (2015).
